# CD4^+^CD25^+^ regulatory T cells ex vivo generated from autologous naïve CD4^+^ T cells suppress EAE progression

**DOI:** 10.1038/s41598-024-56739-2

**Published:** 2024-03-15

**Authors:** Ting-Ting Yang, Pen-Ju Liu, Qing-Yu Sun, Ze-Yi Wang, Guo-Bin Yuan, Ze-Xin Fan, Lin Ma, Jian-Feng Lu, Bo-Yi Yuan, Wen-Long Zou, Li-Min Zhao, Qian Li, Guang-Zhi Liu

**Affiliations:** 1 Department of Neurology, Beijing Anzhen Hospital, Capital Medical University, Beijing, China; 2 Department of Anesthesiology, Chang Hai Hospital, Naval Military Medical University, Shanghai, China; 3 Experimental Center, Beijing Institute of Heart Lung and Blood Vessel Diseases, Beijing, China; 4 Department of Biochemistry and Molecular Biology, School of Basic Medical Sciences, Capital Medical University, Beijing, China

**Keywords:** Multiple sclerosis, Experimental autoimmune encephalomyelitis, Antigen specific, Regulatory T cells, Adoptive cell therapy, Cell biology, Neuroscience, Diseases, Medical research, Neurology

## Abstract

CD4^+^CD25^+^ regulatory T cells (Tregs) play an important role in maintaining immune homeostasis in multiple sclerosis (MS). Hence, we aimed to explore the therapeutic efficacy and safety of adoptive cell therapy (ACT) utilizing induced antigen-specific Tregs in an animal model of MS, that is, in an experimental autoimmune encephalomyelitis (EAE) model. B cells from EAE model that were activated with soluble CD40L were used as antigen-presenting cells (APCs) to induce the differentiation of antigen-specific Tregs from naïve CD4 precursors, and then, a stepwise isolation of CD4^+^CD25^high^CD127^low^ Tregs was performed using a flow sorter. All EAE mice were divided into Treg-treated group (2 × 10^4^ cells in 0.2 mL per mouse, n = 14) and sham-treated group (0.2 mL normal saline (NS), n = 20), which were observed daily for clinical assessment, and for abnormal appearance for 6 weeks. Afterward, histological analysis, immunofluorescence and real-time PCR were performed. Compared to sham-treated mice, Treg-treated mice exhibited a significant decrease in disease severity scores and reduced inflammatory infiltration and demyelination in the spinal cord. Additionally, Tregs-treated mice demonstrated higher CCN3 protein and mRNA levels than sham-treated mice. The results of this preclinical study further support the therapeutic potential of this ACT approach in the treatment of MS.

## Introduction

Multiple sclerosis (MS) is a chronic, inflammatory, and demyelinating disease of the central nervous system (CNS) that usually affects young adults aged between 20 and 40 years^1,2^. MS is characterized by focal lymphocytic CNS infiltration, leading to myelin damage and axonal loss, which consequently leads to various neurological deficits and permanent disability^3^. Although there is substantial uncertainty regarding the etiology of MS, it is universally accepted that T cell-mediated autoimmune responses targeting brain myelin underly the pathogenesis of this disorder.

As master regulators of immune responses, regulatory T cells (Tregs), particularly CD4^+^CD25^+^ Tregs, play important roles in halting excessive immune activation and maintaining immune homeostasis^4^. Because of their roles as immune modulators, the therapeutic utilization of Tregs to regulate abnormal immune responses and to promote tolerance in transplantation, autoimmunity, and chronic inflammatory disorders has been widely studied^5–7^. Treg-based immunotherapies using Tregs that are isolated ex vivo or expanded in vitro have been used in the clinic for several diseases, including graft-versus-host disease (GvHD) after allogeneic hematopoietic stem cell transplantation (HSCT)^8^, Crohn’s disease^9^, uveitis^10^, and early onset type 1 diabetes (T1DM)^11,12^.

In the field of MS, a few studies on adoptive cell therapy (ACT) using Tregs to treat animal models of MS and experimental autoimmune encephalomyelitis (EAE) have been performed, mainly owing to the immunosuppressive properties of Tregs. Interestingly, CCN3, a matricellular protein, has also been reported to mediate Tregs-driven differentiation of oligodendrocyte progenitor cells (OPCs) and CNS myelination, thus implicating a regenerative role for Tregs in CNS remyelination^13^. However, these expanded CD4^+^CD25^+^ Tregs are polyclonal when stimulated with a CD28 superagonist in vivo or concurrently with a CD3/CD28 monoclonal antibody (MoAb) plus high-dose interleukin (IL)-2 in vitro^14,15^. Notably, in a recent, small-scale phase I clinical trial (n = 14), polyclonal CD4^+^CD25^high^CD127^−^FoxP3^+^ Tregs were administered to treat relapsing–remitting MS^16^. This study reported no serious adverse events in the participants, but some of the participants experienced clinical deterioration during follow-up. Despite these promising results, there is accumulating evidence from animal models that antigen-specific Tregs are more potent than polyclonal Tregs in suppressing pathological immune responses in a disease-specific manner^17–23^. This is probably because antigen-specific Tregs predominantly localize to the site of antigen presentation, thus lowering the risk of immunosuppression and immunotherapy-related adverse effects^12^. Additionally, lower cell numbers are required for antigen-specific Tregs therapy than for polyclonal therapeutic approaches due to increased Tregs migration to target tissues. It is potentially feasible to obtain these cell numbers using standard in vitro expansion methods^24^. Hence, antigen-specific Tregs may be more effective and safer than nonselected polyclonal Tregs for ACT. On the other hand, various human studies have reported impaired immune activities of CD4^+^CD25^+^ Tregs derived from MS patients, rather than decreased Tregs numbers^25–28^, thereby providing another advantage of using antigen-specific Tregs for MS treatment.

To investigate this issue, we recently developed a unique approach using allogeneic soluble CD40L (sCD40L)-activated B cells as antigen-presenting cells (APCs) to induce the differentiation of human myelin antigen-specific Tregs from naïve CD4^+^ T cells. As a result, this approach yielded more CD4^+^CD25^+^ Tregs with stronger immunoactivity^29^. Nevertheless, there is still a great need for further preclinical studies on the use of antigen-specific Treg-based therapies in EAE models before these therapies are investigated in clinical trials. Hence, in the present study, a mouse model of EAE was utilized to determine the therapeutic effect of antigen-specific Tregs on EAE symptoms, EAE-related spinal cord pathology, and proinflammatory cytokine and CCN3 expression to provide more evidence about this immunotherapeutic approach for MS.

## Materials and methods

### Animals

Eight- to ten-week-old female wild-type (WT) *C57BL/6* mice weighing 18–20 g were purchased from Beijing HFK Bio-Technology Company (Beijing, China). All the mice were maintained under specific pathogen-free conditions in the animal facility of Beijing Anzhen Hospital (Beijing, China). The mouse study protocol was approved by the Capital Medical University Animal Care and Use Committee (Protocol Number: AEEI-2019-100). This study was reported in accordance with ARRIVE guidelines (https://arriveguidelines.org). The animal treatments were carried out in accordance with the National Institute of Health’s Guide for the Care and Use of Laboratory Animals, and 5 mice were housed in each cage. All mice were euthanized by deeply anesthetizing with 1% pentobarbital.

### Establishment of the EAE model

According to a previously established method^30^, WT *C57BL/6* mice were subcutaneously immunized with 200 μg of murine myelin oligodendrocyte glycoprotein (MOG)_35–55_ peptide (Catalog Number: MOG-001, Chinese Peptide Co., Ltd., Hangzhou, China) in complete Freund’s adjuvant (Catalog Number: 7001, Chondrex Inc., Woodinville, WA, USA). Each mouse was intraperitoneally injected with 400 ng of pertussis toxin (Catalog Number: 181, List Biological Laboratories, Campbell, CA, USA) twice at 0 h and 48 h postimmunization. Clinical assessment of EAE was conducted daily after immunization with the MOG_35–55_ peptide, and disease severity was scored using established criteria as previously described^31^: (1) 0 = normal; (2) 1 = limp tail; (3) 2 = weakness or partial paralysis of two hind limbs; (4) 3 = complete paralysis of two hind limbs; (5) 4 = paralysis of both fore limbs and hind limbs; and (6) 5 = moribund. In addition, healthy WT *C57BL/6* mice that were immunized with normal saline (NS) in complete Freund’s adjuvant were included in the sham (control) group (n = 10). In the present study, EAE mice were sacrificed at two time points: first, on day 14–18 postimmunization for separation and culture of B cells as well as isolation of naïve CD4^+^ T cells, for further in vitro induction of antigen-specific Tregs; second, at 6 weeks posttreatment the EAE mice which obtained the intravenous administration of the antigen-specific Tregs, and were subjected to observation, additional histological analysis, immunofluorescence and real-time PCR.

### Separation and culture of allogeneic B cells

All for this purpose mice were sacrificed on 14–18 days postimmunization, due to the obvious symptoms of EAE during the symptomatic phase of this disease. The lymph nodes and spleen from EAE mice (allogeneic setting) were mechanically homogenized, and then passed through a 70 μm cell strainer. Mononuclear cells (MNCs) were isolated from the cell suspension by Ficoll gradient centrifugation. B cells were isolated from MNCs using a B cell magnetic bead separation kit (Catalog Number: 130-090-862, Miltenyi Biotec, Bergisch Gladbach, NRW, Germany). The purity of the B cells was measured by anti-B220 MoAb (Catalog Number: 553089, BD) staining and flow cytometry, and only cells with purity > 90% were used for further experimentation. Isolated B220^+^ B cells were resuspended in cell culture media (Catalog Number: 100-0646, STEMCELL Technologies Canada Inc., Vancouver, BC, Canada) and incubated with 1 μg/mL megaCD40L (Catalog Number: ALX-522-120-C010, Enzo Life Sciences, Inc. Farmingdale, NY, USA) and 2 μg/mL interleukin (IL)-4 (Catalog Number: 214-14, PeproTech, Rocky Hill, NJ, USA) in the presence of 20 μg/mL MOG_35-55_ peptide at 37 °C in 5% CO_2_ for 7–10 days. The cell culture media were changed every 2–3 days. Four-color staining was performed by incubating 3 × 10^5^ cells with allophycocyanin (APC)-conjugated anti-mouse CD80 (Catalog Number: 560016), brilliant violet (BV) 421-conjugated anti-mouse CD86 (Catalog Number: 564198), phycoerythrin (PE)-conjugated anti-mouse B220 and fluorescein isothiocyanate (FITC)-conjugated anti-mouse MHC-II MoAb (Catalog Number: 562009, BD Biosciences, San Jose, CA, USA) antibodies at room temperature (RT) for 30 min. The cells were then washed twice in PBS and analyzed using flow cytometry. The viability of the B cells was measured using Annexin V-PI (Catalog Number: 556547, BD Biosciences, San Jose, CA, USA) staining and flow cytometry; then, the B cells were further cocultured with naïve CD4^+^ T cells as APCs to induce CD4^+^CD25^+^ Tregs.

### Isolation of naïve CD4^+^ T cells

The same mice were used for isolation of naïve CD4^+^ T cells, which were purified from isolated autologous MNCs from EAE model mice using a CD4^+^ T cell magnetic bead separation kit (Catalog Number: 130-104-453, Miltenyi Biotec). The purity of the naïve CD4^+^ T cells was determined using peridinin chlorophyll protein (PerCP)-conjugated anti-mouse CD4 (Catalog Number: 550954), PE-conjugated anti-mouse CD25 (Catalog Number: 101904), and APC-conjugated anti-mouse CD127 MoAb (Catalog Number: 564175, BD Biosciences, San Jose, CA, USA) antibodies and a flow sorter, and only cells with purity > 90% were used for further experimentation; then, the naïve CD4^+^ T cells were further cocultured with the sCD40L-activated B cells to induce CD4^+^CD25^+^ Tregs.

### Induction, expansion and separation of CD4^+^CD25^high^CD127^low^ Tregs

Similarly, next, isolated naïve CD4^+^ T cells were cocultured with allogeneic sCD40L-activated B cells at a ratio of 10 to 1 in X-vivo15 medium (Catalog Number: 04-418Q, Lonza Inc., Basel, Switzerland) supplemented with 5% heat-inactivated fetal bovine serum (FBS) (Catalog Number: 10099141, Gibco, Carlsbad, CA, USA) for 14 days in the presence of 100 unit/mL IL-2 (Catalog Number: 212-12, PeproTech, Rocky Hill, NJ, USA) and 20 μg/mL murine MOG_35–55_ peptide as described in our previous study^29^. The cells were harvested on day 14, followed by isolation of CD4^+^CD25^high^CD127^low^ Tregs with PerCP-conjugated anti-mouse CD4, PE-conjugated anti-mouse CD25, and APC-conjugated anti-mouse CD127 MoAbs and a fluorescence-activated cell sorter (Becton Dickinson, San Jose, CA, USA); then, the CD4^+^CD25^+^ cells were further adoptively transferred to EAE mice for the treatment of this disease.

### Detection of forkhead box protein 3 by FACS

To determine cell bioactivities, intracellular expression of forkhead box protein 3 (Foxp3) were measured by FACs. Briefly, suspensions of MNCs or induced MOG-reactive CD4^+^CD25^+^ Tregs were prepared and incubated with PerCP-conjugated anti-mouse CD4 and PE-conjugated anti-mouse CD25 MoAbs (BD Biosciences, San Jose, CA, USA) for 30 min at RT. After washing twice with Fixation/Permeabilization buffer (Catalog Number: 00-5523-00, Thermo, Waltham, MA, USA), the cells were incubated with Alexa Fluor647-conjugated anti-mouse forkhead box protein 3 (Foxp3) MoAb (Catalog Number: 560402, BD Biosciences, San Jose, CA, USA) for 30 min at RT. The cells were then washed with staining buffer and analyzed using flow cytometry (FACScan, BD Biosciences, San Jose, CA, USA) and BD CellQuest™ Pro software (© 2002, BD Biosciences, San Jose, CA, USA).

### Adoptive transfer of antigen-specific CD4^+^CD25^+^ Tregs

Twelve days after the establishment of the EAE model using the same method as mentioned in establishment of the EAE model section, the stochastic indicator method was used to randomly divide all EAE mice into two groups: the Treg-treated group (2 × 10^4^ cells in 0.2 mL per mouse, n = 14) and the sham-treated group (0.2 mL NS per mouse, n = 20). In addition, healthy WT *C57BL/6* mice that were simultaneously administered the same volume of NS (0.2 mL per mouse) were included as the WT group (n = 14). Tregs or NS were intravenously administered to EAE or WT mice accordingly. After the administration of CD4^+^CD25^+^ Tregs or NS, all the mice underwent daily clinical assessment to monitor EAE, progression and abnormal appearance (e.g., fever, local skin change). All the mice were sacrificed at 6 weeks posttreatment, and samples were harvested for additional histological analysis, real-time PCR, complete blood counts, and blood biochemical analyses. All outcomes were rated by independent evaluators who were blinded to the treatment assignment.

### Histological analysis

For pathological analyses, after anesthetization with 1% pentobarbital, mice were subjected to perfusion through the left ventricle first with NS to clear the blood and then with 4% paraformaldehyde, and tissues from the lumbar segments of spinal cords were embedded in paraffin. Once 5-μm-thick sections had been deparaffinized and rehydrated, they were stained with hematoxylin and eosin (H&E) or Luxol fast blue (LFB) to assess inflammation and demyelination. The 360 H&E images and 72 LFB images were acquired on a Nikon Eclipse Ni-E microscope (Nikon Corp., Tokyo, Japan). Three sections were selected from every spinal cord segment, and five high-power fields in each section were chosen for H&E observation. The degree of inflammatory cell infiltration into the spinal cord was scored using previously published criteria^32^: 0, no inflammatory cell infiltration; 1, inflammatory cell infiltration was limited to areas around the perivascular and spinal meninges; 2, mild inflammatory cell infiltration into the parenchyma of the spinal cord (1–10 cells/field); 3, moderate inflammatory cell infiltration into the parenchyma of the spinal cord (10–100 cells/field); and 4, heavy inflammatory cell infiltration into the parenchyma of the spinal cord (> 100 cells/field). The percentage of demyelinated area was measured by manually outlining the total white matter that was stained with LFB using ImageJ software (https://imagej.nih.gov/ij/). The demyelinating ratio is calculated by dividing the demyelinating area by the total white matter area.

### Immunofluorescence

For the immunofluorescence assay, spinal cord sections were blocked with 1% FBS in PBS for 1 h and incubated with goat anti-Olig2 (Catalog Number: AF2418, 15 μg/mL, R&D Systems, Minneapolis, MN, USA) and rabbit anti-CCN3 antibodies (Catalog Number: 8767S, Cell Signaling Technology, Boston, MA, USA) at 1/200 dilution overnight at 4 °C. The specificity of these two primary antibodies was determined by testing in at least one approved application (e.g., western blotting) as detailed in the datasheets. The sections were washed with PBS, treated with Alexa Fluor 488 goat anti-rabbit IgG [Catalog Number: A11034, Thermo Fisher Scientific, Waltham, MA, USA] and Alexa Fluor 594 donkey anti-goat IgG secondary antibodies [Catalog Number: A11058, Thermo Fisher Scientific, Waltham, MA, USA]), and then incubated for 1 h at RT. Finally, 4ʹ,6-diamidino-2-phenylindole, dihydrochloride (DAPI) (Catalog Number: H-1200-10, Vector, USA) nuclear staining was performed to quantify cell numbers. One section was selected from every spinal cord segment, and two regions were selected in each section (Fig. 4E). The images of histopathological sections were acquired on a KFBio KF-PRO-005 digital scanner (KFBio, Ningbo, China), and converted to gray scale images for further cell characterization and quantification using ImageJ software (https://imagej.nih.gov/ij).

### RNA preparation and cDNA synthesis

For real-time PCR, tissues from the lumbar segments of the spinal cord were harvested from the mice, which were anesthetized with 1% pentobarbital and perfused with NS. TRIzol (1 mL/50–100 mg tissue) was added to the lumbar spinal cord samples, and total RNA was isolated using chloroform. Reverse transcription was performed using the Reverse Transcription System (Catalog Number: A5001, Promega, Madison, WI, USA). Samples were incubated with random primers at 70 °C for 5 min, and then, they were mixed with the recombinant Rnasin Ribonuclease inhibitor, reverse transcriptase, PCR nucleotide Mix, reaction buffer and MgCl_2_. The reactions were carried out at 25 °C for 5 min and 42 °C for 60 min using a C1000 Thermal Cycler (Bio-Rad, Hercules, CA, USA).

### Real-time PCR

Real-time PCR was performed in a total volume of 20 μL, which included 2 μL cDNA template, 250 nM of each primer and 10 μL Taq Pro Universal SYBR qPCR Master Mix (Catalog Number: Q712-03, Nanjing Vazyme Biotech Co., Nanjing, China). Primers were synthesized by Sangon Biotech (Shanghai) using previously published sequences (https://pga.mgh.harvard.edu/primerbank/) (Table 1). PCR was performed using a CFX96 Fast Sequence Detector (Bio-Rad, Hercules, CA, USA). All the reactions were performed in triplicate. Reactions were performed at 95 °C for 10 min, followed by 42 cycles of 15 s at 95 °C, and 1 min at 60 °C. The gene expression levels were calculated by using the 2^–ΔΔCT^ (comparative threshold cycle, or CT) method, as described by the manufacturer (Technical Bulletin 2; Applied Biosystems).

**Table 1:** Primers used in this study.

Gene	Sense primer (5ʹ–3ʹ)	Antisense primer (5ʹ–3ʹ)
Ifn-γ	CTGGCAGGATGATTCTGCTGG	GCATACGACAGGGTTCAAGTTAT
Il-6	TAGTCCTTCCTACCCCAATTTCC	TTGGTCCTTAGCCACTCCTTC
Tnf	TCTTCTCATTCCTGCTTGTGG	GGTCTGGGCCATAGAACTGA
Il-10	CACTGCTATGCTGCCTTCTCT	TGTCTTCCCTGCTGTACTGT
Ccn3	CGATGGGGTCATTTACCGCAA	TGCGACTTTTCTCGGAGCTG
Gapdh	AAATGGTGAAGGTCGGTGTGAAC	CAACAATCTCCACTTTGCCACTG

### Statistical analysis

Sample size calculation in this study was performed online (http://powerandsamplesize.com/) using a power analysis based on one-way ANOVA pairwise, two-sided equality. Considering a significance criterion of *α* = 0.025 and power = 0.90, a sample size of 12 was needed. Thus, the obtained sample size of *n* = 14–20 for the therapeutic window studies was sufficient to account for accidental loss.

The data are expressed as the mean ± standard deviation (SD) or median and interquartile range. Normality and variance homogeneity were assessed using the Kolmogorov–Smirnov test. One-way ANOVA followed by multiple comparisons with Bonferroni calibration was performed if the data were normally distributed and consistent with homogeneity of variance (CCN3^+^Olig2^+^ cells, CCN3^+^DAPI^+^ cells or proportion of CCN3^+^Olig2^+^ cells, Il-10 mRNA level). Kruskal‒Wallis nonparametric tests were performed for comparisons of nonnormally distributed data (inflammatory scores, percent demyelination, and Tnf, Il-6, Ccn3 and Ifn-γ mRNA levels). A generalized linear mixed model (GLMM) was used to compare longitudinal data in the same biological replicates (clinical scores and body weight), if the data were not normally distributed. All the statistical analyses were performed with SPSS software (version 29.0), and the data were graphed with the GraphPad Prism 9 package. A P-value < 0.05 was considered statistically significant.

### Ethics approval

Animal treatments were conducted in accordance with the National Institute of Health’s Guide for the Care and Use of Laboratory Animals. Approval was granted by the Capital Medical University Animal Care and Use Committee.

## Results

### Mice developed EAE symptoms on 9 days postimmunization

To confirm whether the EAE model was successfully established, we observed EAE symptoms after immunization with the MOG_35–55_ peptide. On 9 days postimmunization, *C57BL/6* mice presented with mild symptoms that became obvious on day 11, peaked on day 16 and stabilized thereafter, with a median maximum score of 2.5 (1.0) (Fig. S1A, Table S4). Mice with scores of 1–4 were utilized for the following experiments. No mice in the sham group developed EAE. Moreover, the body weights and clinical scores changed significantly over time (Table S3). The EAE group displayed lower body weights than the sham group, particularly on day 16 postimmunization (18.10 (2.55) g vs. 23.65 (2.70) g, *P* < 0.001; Fig. S1B, Table S4).

### MOG-reactive CD4^+^CD25^high^CD127^low^ Tregs were induced and expanded from naïve CD4^+^ T cells in the presence of sCD40L-activated B cells

**Figure 1:**
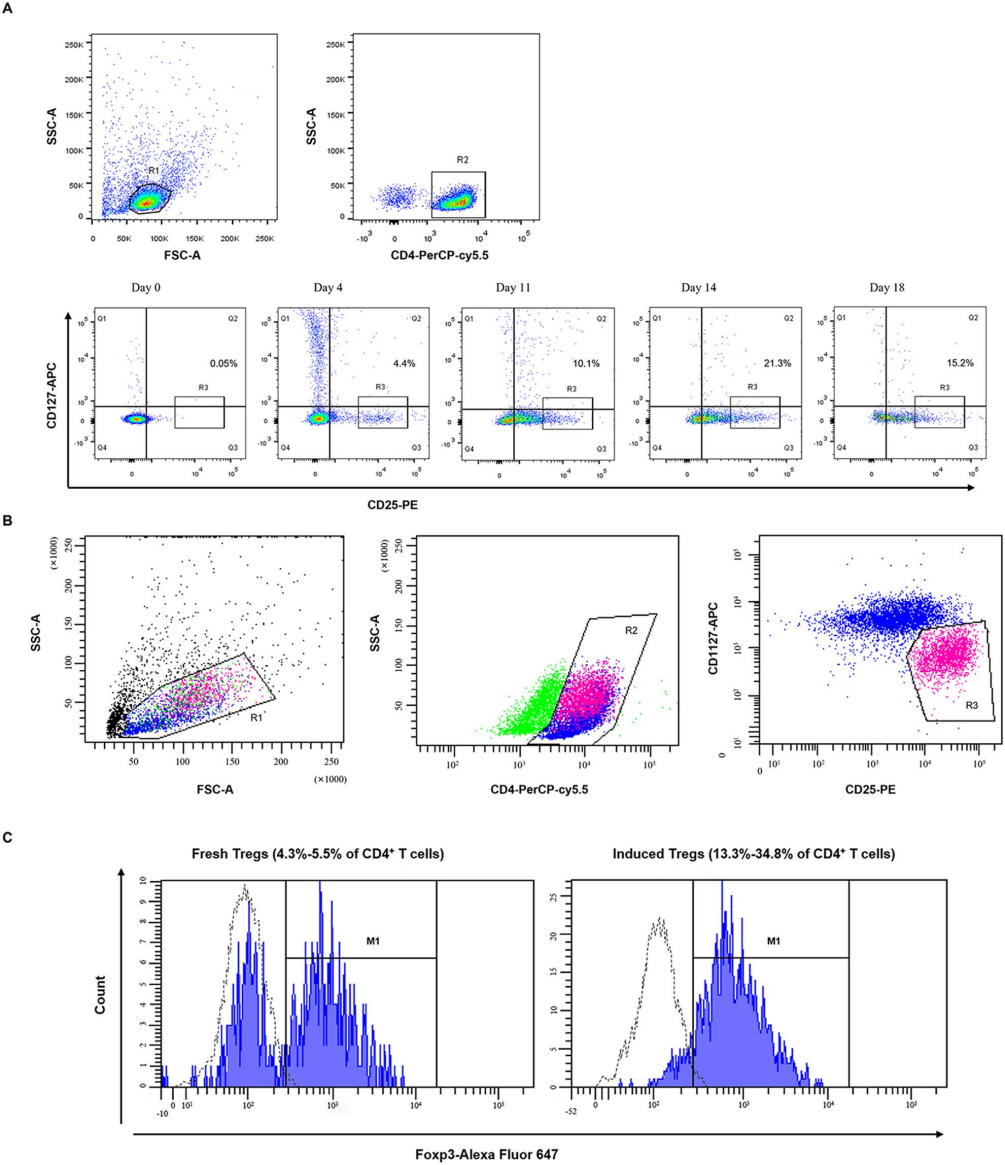
MOG-reactive CD4^+^CD25^high^CD127^low^ regulatory T cells (Tregs) were induced and expanded from naïve CD4^+^ T cells in the presence of sCD40L-activated B cells, and demonstrated higher Foxp3 expression. (**A**) Autologous naïve CD4^+^ T cells (0.5–1 × 10^6^ cells) were induced to differentiate into MOG-reactive CD4^+^CD25^high^CD127^low^ Tregs after 4, 11, 14 and 18 days of coculture with allogeneic sCD40L-treated B cells and the MOG_35–-55_ peptide. (**B**) Isolation of CD4^+^CD25^high^CD127^low^ Tregs was performed with PerCP-conjugated anti-CD4, PE-labeled anti-CD25, and APC-conjugated anti-CD127 monoclonal antibodies (MoAbs) and a flow sorter. Region 1 (R1) was selected to set the mononuclear cell gate according to forward light scatter (FSC) and side light scatter (SSC) properties, and region 2 (R2) was further selected to set the CD4^+^ T-cell gate. Region 3 (R3) was selected to set the third gate to separate CD4^+^CD25^high^CD127^low^ Tregs. Control staining with isotype control antibodies was utilized to define the gates. (**C**) Forkhead box protein 3 (Foxp3) expression in fresh CD4^+^CD25^+^ Tregs (4.3–5.5% of CD4^+^ T cells) and induced MOG-reactive CD4^+^CD25^high^CD127^low^ Tregs (13.3–34.8% of CD4^+^ T cells) was measured. Dotted line histogram represents the isotype control antibody, which was used as control to define the gate.

To investigate the dynamic changes in the in vitro differentiation and expansion as well as the frequency of induced antigen-specific Tregs, naïve CD4^+^ T cells were induced to differentiate into MOG-reactive CD4^+^CD25^high^CD127^low^ Tregs after coculture with sCD40L-activated B cells and the MOG_35-55_ peptide. As shown in Fig. 1A, the proportion of CD4^+^CD25^high^CD127^low^ Tregs demonstrated a trend of continuously increasing at 4, 11, and 14 day post coculture and then showed a trend of decreasing at 18 day post coculture (Fig. 1A). Induced Tregs were finally acquired after 14 day of coculture via a fluorescence-activated cell sorter using anti-CD25, anti-CD4, and anti-CD127 MoAbs (Fig. 1B). As a result, the frequency of CD4^+^CD25^high^CD127^low^ Tregs among CD4^+^ T cells was 13.3–34.8% (Table 2).

**Table 2:** Frequency of induced CD4^+^CD25^high^CD127^low^ regulatory T cells (Tregs) in five individual experiments from EAE mice (n = 5).

Subjects	Frequency of Tregs in CD4^+^ T cells (%)
1	26.2
2	34.8
3	28.6
4	21.3
5	13.3

### Induced MOG-reactive CD4^+^CD25^high^CD127^low^ Tregs demonstrated higher Foxp3 expression

FACS was used to detect Foxp3 expression in freshly isolated CD4^+^CD25^+^ Tregs and induced antigen-specific Tregs. As shown in Fig. 1C, 60% of freshly isolated CD4^+^CD25^+^ Tregs expressed Foxp3, whereas 90% of induced MOG-reactive CD4^+^CD25^+^ Tregs expressed Foxp3. In addition, > 95% of both populations were CD4^+^CD25^high^CD127^low^.

### Induced MOG-reactive CD4^+^CD25^high^CD127^low^ Tregs ameliorated EAE symptoms

**Figure 2:**
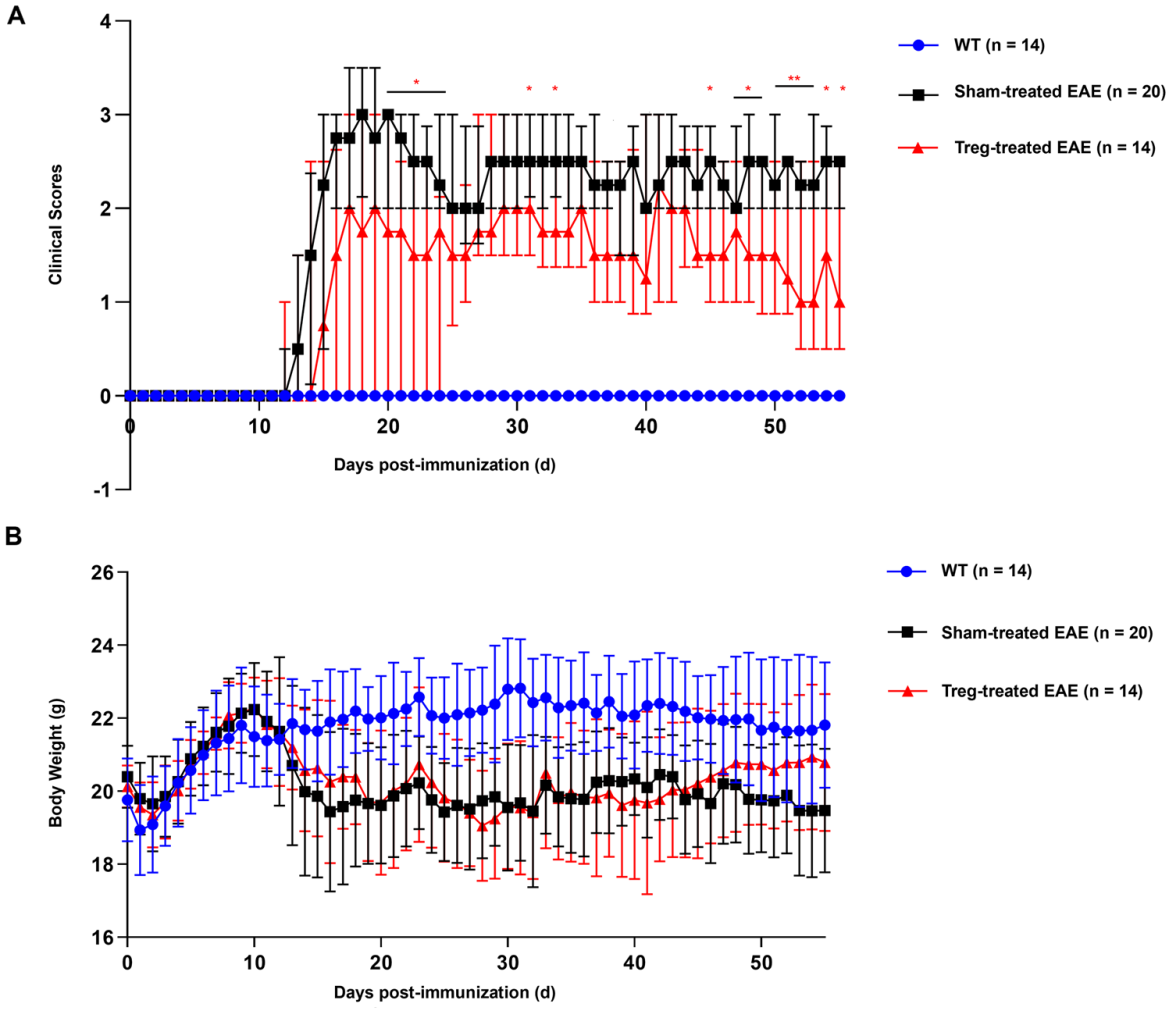
Induced MOG-reactive CD4^+^CD25^high^CD127^low^ regulatory T cells (Tregs) ameliorated experimental autoimmune encephalomyelitis (EAE) symptoms. Changes in (**A**) disease severity scores and (**B**) body weight was observed in the wild-type (WT) (n = 14), sham-treated EAE (n = 20), and Treg-treated EAE groups (n = 14). All the data are presented as the median and interquartile range. *P < 0.05, **P < 0.01.

Disease severity and body weight were measured dynamically to determine the impact of induced antigen-specific Tregs on suppressing EAE. As expected, the Treg-treated group showed a significant decrease in disease severity scores compared to the sham-treated group, particularly on day 50 [2.25 (1.00) vs. 1.50 (1.60), *P* < 0.01], 51 [2.50 (0.50) vs. 1.25 (1.60), *P* < 0.01], 52 [2.25 (0.50) vs. 1.00 (2.00), *P* < 0.01], and 53 [2.25 (1.00) vs. 1.00 (2.00), *P* < 0.01] (Fig. 2A). Moreover, the Treg-treated group and sham-treated group demonstrated lower body weights than the WT group, and tended to recover at 5–6 weeks post treatment. However, no remarkable difference in body weight was observed between the Treg- and sham-treated groups (Fig. 2B).

### Induced MOG-reactive CD4^+^CD25^high^CD127^low^ Tregs improved EAE-associated inflammation and demyelination in the spinal cord

**Figure 3:**
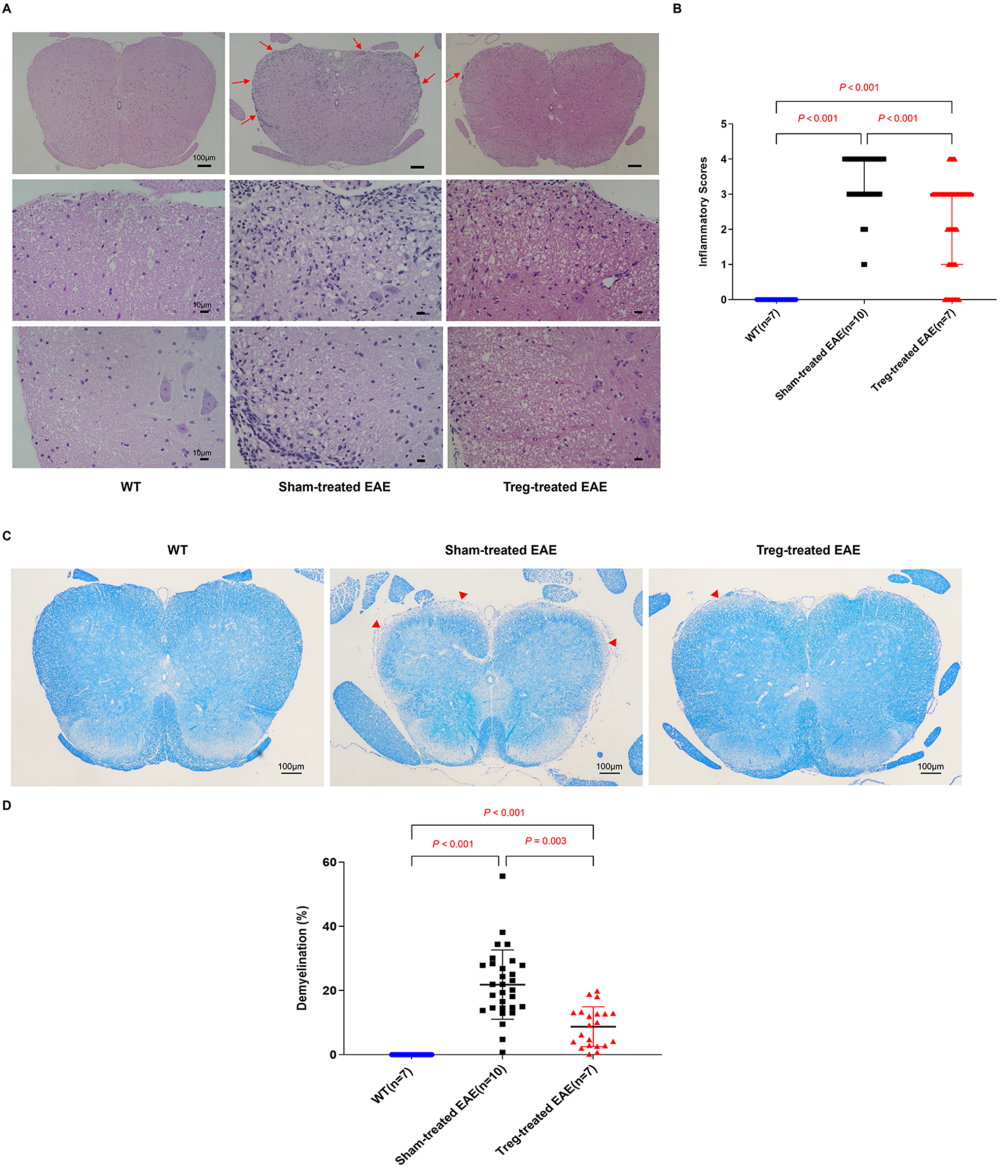
Induced MOG-reactive CD4^+^CD25^high^CD127^low^ regulatory T cells (Tregs) improved experimental autoimmune encephalomyelitis (EAE)-associated inflammation and demyelination in the spinal cord. (**A**) Histological analysis of lumber segments was performed using hematoxylin and eosin (H & E) staining. Five different regions (H & E, × 400) in each section of three sections in the lumbar segment of the spinal cord (H & E, × 40) were selected for further analysis. The wild-type (WT) group (n = 7) demonstrated no inflammatory cell infiltration, and reduced inflammatory infiltration (long red arrow) was present in the regulatory T cell (Treg)-treated group (n = 7) compared with the sham-treated group (n = 10), as shown by (**B**) a comparison of the scores for the degree of inflammatory cell infiltration into the spinal cords among the three groups. (**C**) Histological analysis of three sections in each lumber segments was performed using Luxol fast blue (LFB) staining (LFB, × 40). The wild-type (WT) group (n = 7) demonstrated no demyelination, whereas decreased demyelination (short red arrow) was present in the regulatory T cell (Treg)-treated group (n = 7) compared with the sham-treated group (n = 10), as shown by (**D**) a comparison of the pixel area (%) of demyelination in total white matter among the three groups. All the data are presented as the median and interquartile range.

H&E and LFB staining were utilized to determine the effect of induced antigen-specific Tregs on EAE-related pathology. The Treg-treated group had lower inflammatory cell infiltration (revealed by H&E staining) in the lumbar segments than the sham-treated group [3.00 (2.00) vs. 4.00 (1.00), *P* < 0.001] (Fig. 3A,B). LFB staining revealed reduced demyelination [9.22 (10.13) % vs. 21.04 (13.42) %, *P* = 0.003] in the lumbar segment of the Treg-treated group compared with that in the sham-treated group (Fig. 3C,D). In addition, neither inflammatory cell infiltration nor demyelination was observed in the WT group.

### Induced MOG-reactive CD4^+^CD25^high^CD127^low^ Tregs upregulated intracellular expression of CCN3 in the spinal cord

**Figure 4:**
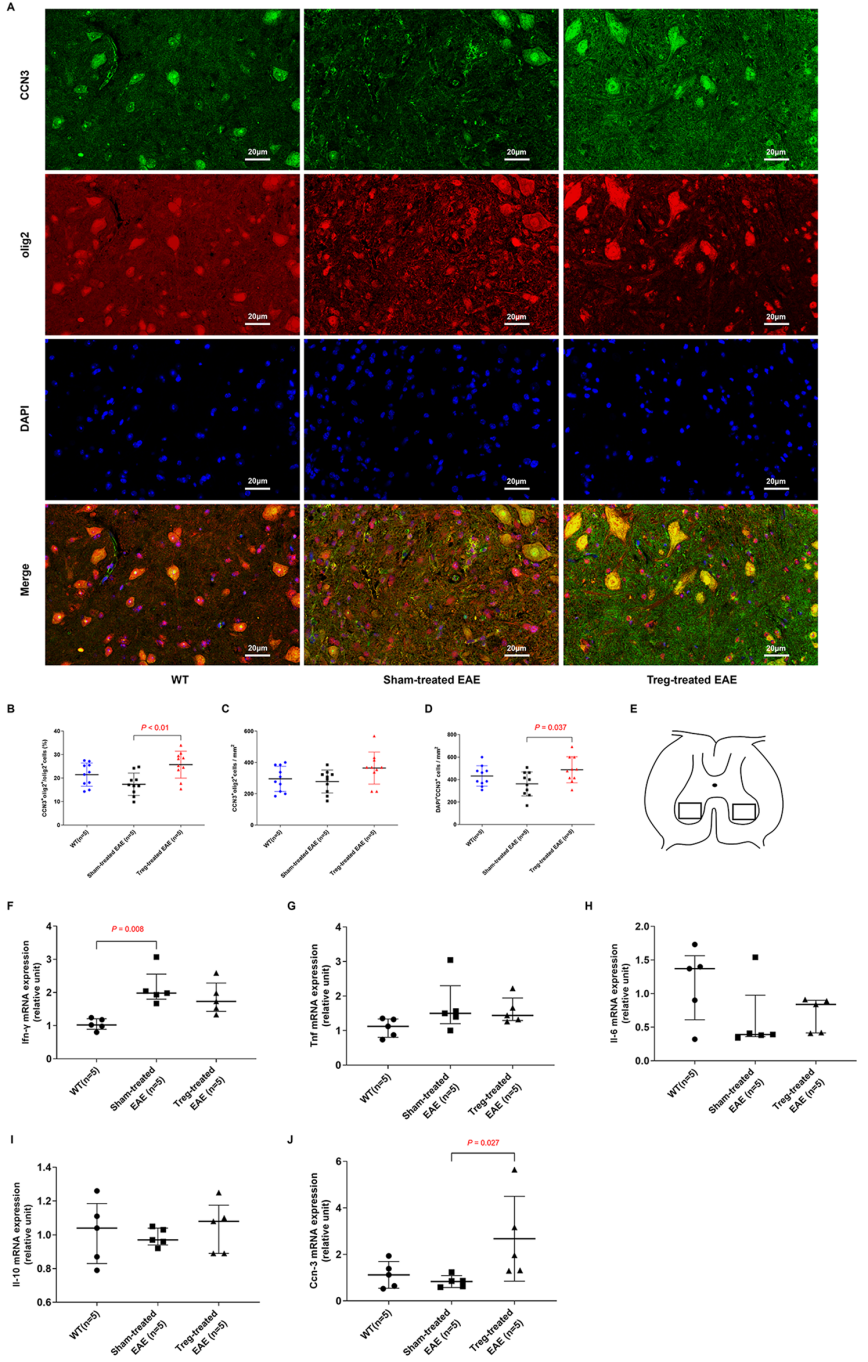
Induced MOG-reactive CD4^+^CD25^high^CD127^low^ regulatory T cells (Tregs) upregulated intracellular expression of CCN3 and Ccn3 mRNA expression in the spinal cord. (**A**) Immunofluorescence analysis of lumber segments was performed to determine the localization of intracellular CCN3 (× 400). The Treg-treated group (n = 5) displayed marked upregulation of CCN3 in the spinal cord compared to the sham-treated group (n = 5), as shown by (**B–D**) a comparison of the number of CCN3^+^olig2^+^ cells (cells/mm^2^) or proportion of CCN3^+^Olig2^+^ cells (%) in the spinal cords among the groups. (**E**) The schematic diagram depicts the location of the micrographs. All the data are presented as the mean ± SD. Interferon (Ifn)-γ, tumor necrosis factor (Tnf), interleukin (Il)-6, Il-10, and Ccn3 mRNA levels (**F–J**) in the spinal cords were compared among the Treg, control, and sham groups. Il-10 data are presented as the mean ± SD, while other data are presented as the median and interquartile range.

In a recent study by Dombrowski et al., CCN3 was found to be involved in Treg-mediated oligodendrocyte differentiation and CNS myelination^13^. Hence, we performed an immunofluorescence assay to investigate the intracellular expression of CCN3 after the adoptive transfer of induced antigen-specific Tregs. Two high-power fields in each section were chosen for immunofluorescence observation (Fig. 4E). Compared to the sham-treated group, the Treg-treated group demonstrated significantly increased number of CCN3^+^ cells or proportions of CCN3^+^Olig2^+^ cells in lumbar segments (361.7 ± 107.0 cells/mm^2^ vs. 487.6 ± 115.7/mm^2^, *P* = 0.037; 17.37 ± 4.76% vs. 25.72 ± 5.74%, *P* < 0.01). However, no marked difference in either CCN3 expression or the proportion of CCN3^+^Olig2^+^ cells was found between the Treg-treated and WT groups (Fig. 4A,B,D). Although higher numbers of CCN3^+^Olig2^+^ was observed in the lumbar segments of Treg-treated mice than in those of either sham-treated mice or WT mice, the difference among these three groups did not reach statistical significance (Fig. 4C).

### Induced MOG-reactive CD4^+^CD25^high^CD127^low^ Tregs upregulated Ccn3 mRNA expression in the spinal cord

To further measure cytokine and CCN3 expression at the transcription level, real-time PCR was performed to quantify the mRNA expression of these molecules. In the spinal cord, no significant difference in Tnf, Ifn-γ, Il-6, and Il-10 mRNA expression was observed between the Treg-treated group and the sham-treated group, except for higher Ifn-γ mRNA expression in the sham-treated group than that in the WT group (*P* < 0.01) (Fig. 4F–I). However, there was an increase in the Ccn3 mRNA levels in the Treg-treated group compared to the sham-treated group (*P* < 0.05) (Fig. 4J).

### Adoptive transfer of induced MOG-reactive CD4^+^CD25^high^CD127^low^ Tregs caused no obvious adverse events

None of the mice that received any of therapeutic regimens exhibited any obvious adverse events, such as fever or local skin change. In addition, complete blood counts and blood biochemical analysis after treatment showed no remarkable differences among the three groups (Table S1).

## Discussion

Accumulating evidence has confirmed the important role of CD4^+^CD25^+^ Tregs in preventing or ameliorating spontaneous or induced EAE^14,15,19,33–36^. Among CD4^+^CD25^+^ Treg subsets, the antigen-specific Treg population demonstrated better suppression of CD4^+^ effector T cell (Teff) proliferation and control of CNS myelin-induced EAE in vivo than polyclonal Tregs^19,37,38^. More strikingly, smaller numbers of antigen-specific Tregs (2–10 × 10^4^ cells) than polyclonal Tregs (2–4 × 10^6^ cells) were administered^14,15,19,37^. In the present study, adoptive transfer of 2 × 10^4^ antigen-specific Tregs into recipient EAE model mice resulted in reduced EAE symptoms, reduced CNS inflammation and demyelination. Additionally, a dose of antigen-specific Tregs up to 2 × 10^4^ cells per mouse was well-tolerated, and no obvious adverse effects were observed. Taken together, our results thus confirm the treatment efficacy and safety of this therapeutic regimen in the treatment of EAE.

In general, human CD4^+^CD25^high^CD127^low^ Tregs account for 5–10% of the total CD4^+^ T-cell population in circulation^39,40^, and antigen-specific Tregs comprise a smaller proportion (0.01–0.1%) and thus far have not been effectively expanded^41–43^. Hence, it is crucial to generate adequate numbers of antigen-specific Tregs for the treatment of autoimmune diseases with known target antigens, such as MS, T1DM, and rheumatoid arthritis. In this study, more antigen-specific Tregs were obtained (13.3–34.8%), and they exhibited normal or even enhanced function, since the expression of Foxp3, a key regulator of the development and functional bioactivities of Tregs^44–46^, was observed to be mildly elevated in induced Tregs compared to freshly isolated Tregs. Together with similar results from our previous human Treg study^29^, the findings of this study further support the clinical feasibility of this naïve CD4^+^ T cell-derived Treg protocol for the treatment of MS. On the other hands, Tregs manufacturing in our study is still not ideal, mostly due to the relatively low proliferation rates of Tregs in vitro. Hence, culture media, growth factors and stimulants suitable for Tregs biology and accommodating donor variability are needed to be developed in the future.

Various studies have reported that Tregs suppress autoreactive T cells via multiple mechanisms, such as the secretion of inhibitory cytokines (e.g., IL-10 and transforming growth factor-β [TGF-β]), cell contact-dependent endocytosis (CTLA-4–CD80/CD86), and adenosine-mediated immunosuppression involving the ectoenzymes CD39/CD73^47^. It is currently thought that decreased Treg numbers, impaired Treg suppressive capability, and resistance of pathogenic T cells to Treg control might contribute to the dysregulated T cell-mediated autoimmune responses that are associated with MS pathology^2,48^. Despite the presence of Foxp3^+^ Tregs in acute or chronic demyelinated CNS lesions, Tregs are absent or present in small numbers in MS tissues, probably due to their extremely short-lived nature^49,50^. Indeed, in our study, administration of Tregs did not markedly attenuate the proinflammatory immune response within the CNS of EAE model mice when compared to the control (sham-treated) group; however, these mice exhibited a CNS inflammatory response in the CNS via the release of main cytokines, such as IFN-γ, which is a well-known proinflammatory cytokine that is involved in EAE pathogenesis^51,52^. Therefore, we speculate that antigen-specific Tregs closely regulate the development of autoimmune response in the CNS by regulating autoreactive T cells and maintaining peripheral tolerance toward self-antigens. Nonetheless, more evidence is required to address this issue.

CCN3, also known as nephroblastoma overexpressed [NOV], is a member of the CCN family and has multiple bioactivities that are associated with cell growth, migration, and differentiation^53–55^. In adult mammals, CCN3 is widely expressed in smooth muscle cells, endotheliocytes, fibroblasts, and chondrocytes^56,57^, and it is also expressed in neurons of distinct areas of the nervous system (e.g., dorsal root ganglia, dorsal horn of spinal cord, hippocampus, and central canal), Olig2^+^ and GFAP^+^ glia in the spinal cord^58–60^. Of note, upregulated expression of CCN3 was reported during CNS demyelination and remyelination in a cuprizone-induced mouse model of demyelination^60^, and CCN3 was involved in nonspecific Treg-driven OPCs differentiation and CNS remyelination^13^. In parallel, our study revealed increased Ccn3 mRNA and protein expression in the spinal cords of the antigen-specific CD4^+^CD25^+^ Treg-treated group compared to the sham-treated control group. Additionally, more CCN3^+^ cells were observed in the spinal cords of Treg-treated mice than in sham-treated mice. Taking all these findings into consideration, we confirm for the first time that antigen-specific Tregs can exert neuroprotective effects by upregulating CCN3 expression in oligodendrocytes, ultimately contributing to CNS tissue repair and regeneration.

## Conclusion

In summary, our preliminary preclinical study first showed that the adoptive transfer of a low-dose antigen-specific Tregs efficiently ameliorated EAE symptoms or pathological severity without causing obvious adverse events, and these cells might promote tissue repair and regeneration via the upregulation of CCN3 in the CNS. Hence our findings, to a large extent, confirm the good safety and efficacy of this approach in the treatment of murine EAE and further support the potential use of this ACT approach in MS treatment. An obvious limitation of this study was the limited sample size, and this study did not select different dosages of Tregs or fully elucidate the possible mechanism by which Tregs function in the treatment of EAE. Future investigations using a larger sample size or even clinical trials would help pave the way for the development of this approach into a viable treatment option for MS.

### Supplementary Information

Supplementary Information.

## Data Availability

The datasets used and/or analysed during the current study available from the corresponding author on reasonable request.

## Abbreviations

ACT: Adoptive cell therapy
ANOVA: One-way analysis of variance
APC: Allophycocyanin
APCs: Antigen-presenting cells
BV: Brilliant violet
CNS: Central nervous system
EAE: Experimental autoimmune encephalomyelitis
FBS: Fetal bovine serum
FITC: Fluorescein isothiocyanate
Foxp3: Forkhead box protein 3
GvHD: Graft-versus-host disease
H&E: Hematoxylin and eosin
HSCT: Hematopoietic stem cell transplantation
IFN: Interferon
IL: Interleukin
LFB: Luxol fast blue
MNC: Mononuclear cells
MoAb: Monoclonal antibody
MOG: Myelin oligodendrocyte glycoprotein
MS: Multiple sclerosis
NS: Normal saline
PE: Phycoerythrin
PerCP: Peridinin chlorophyll protein
T1DM: Type 1 diabetes
Teff: Effector T cells
TGF-β: Transforming growth factor-β
TNF: Tumor necrosis factor
Tregs: Regulatory T cells
WT: Wild type
