# Supplementary material for: CD4+CD25+ regulatory T cells ex vivo generated from autologous naïve CD4+ T cells suppress EAE progression
Source: Sci Rep. 2024 Mar 15;14:6262. doi: 10.1038/s41598-024-56739-2 (PMC10943184; doi:10.1038/s41598-024-56739-2)

**Supplementary Material**

**CD4^+^CD25^+^ Regulatory T Cells Generated Ex vivo from Autologous naïve CD4^+^ T Cells Suppress EAE Progression**

***Scientific Reports***

Ting-Ting Yang^1^, Pen-Ju Liu^1^, Qing-Yu Sun^2^, Ze-Yi Wang^1^, Guo-Bin Yuan^1^, Ze-Xin Fan^1^, Lin Ma^1^, Jian-Feng Lu^1^, Bo-Yi Yuan^1^, Wen-Long Zou^1^, Li-Min Zhao^3^, Qian Li^4^, Guang-Zhi Liu^1*^

^1^Department of Neurology, Beijing Anzhen Hospital, Capital Medical University, Beijing, China, ^2^Department of Anesthesiology, Chang Hai Hospital, Naval Military Medical University, Shanghai, China, ^3^Experimental Center, Beijing Institute of Heart Lung and Blood Vessel Diseases, Beijing, China, ^4^Department of Biochemistry and Molecular Biology, School of Basic Medical Sciences, Capital Medical University

***Correspondence:** Guang-Zhi Liu, liugz@mail.ccmu.edu.cn, 100029, +86 13391777952, +86-10-64456119

**Table S1. Complete blood counts and blood chemistry in wild-type (WT), sham-treated experimental autoimmune encephalomyelitis (EAE), and regulatory T cell (Treg)-treated EAE group**

|  | ALT | AST | BUN | CRE | O/P | TBIL | WBC | RBC | HGB | PLT | NEUT# | LYMPH# | MONO# | EO# | BASO# | NEUT% | LYMPH% | MONO% | EO% | BASO% |
| --- | --- | --- | --- | --- | --- | --- | --- | --- | --- | --- | --- | --- | --- | --- | --- | --- | --- | --- | --- | --- |
| WT Group | 22 | 91.2 | 5.8 | 10.9 | 4.145 | 2.05 | 6.03 | 9.04 | 140.0 | 1206 | 0.96 | 3.42 | 0.89 | 0.01 | 0.75 | 15.9 | 56.7 | 14.8 | 0.2 | 12.4 |
|  | 40 | 93.8 | 8.1 | 27.6 | 2.345 | 1.14 | 5.03 | 9.43 | 143.0 | 1281 | 0.21 | 4.36 | 0.22 | 0 | 0.24 | 4.10 | 86.7 | 4.4 | 0 | 4.8 |
|  | 24 | 96.6 | 7.7 | 10.9 | 4.025 | 0.67 | 4.28 | 9.94 | 148.0 | 1159 | 0.22 | 3.79 | 0.02 | 0.01 | 0.24 | 5.10 | 88.6 | 0.5 | 0.2 | 5.6 |
|  | 62 | 135.6 | 9.4 | 18.3 | 2.187 | 1.14 | 6.18 | 10.09 | 152.0 | 1412 | 0.42 | 5.08 | 0.26 | 0 | 0.42 | 6.80 | 82,2 | 4.2 | 0 | 6.8 |
|  | 84 | 228.2 | 11.3 | 15.1 | 2.717 | 1.87 | 6.10 | 9.68 | 153.0 | 919 | 0.11 | 5.44 | 0.12 | 0 | 0.43 | 1.80 | 89.2 | 2.0 | 0 | 7.0 |
|  | 57 | 258.4 | 8.6 | 15.7 | 4.533 | 1.34 | 3.53 | 9.17 | 146.0 | 1143 | 0.38 | 2.64 | 0.09 | 0.12 | 0.30 | 10.8 | 74.8 | 2.5 | 3.4 | 8.5 |
| Sham-treated EAE Group | 27 | 109.7 | 12.8 | 10.9 | 4.063 | 2.54 | 7.50 | 10.08 | 150.0 | 1410 | 0.13 | 6.10 | 0.36 | 0 | 0.91 | 1.8 | 81.3 | 4.8 | 0 | 12.1 |
|  | 55 | 345.1 | 7.7 | 12.2 | 6.275 | 0.91 | 5.01 | 9.20 | 140.0 | 1208 | 0.45 | 2.84 | 1.61 | 0 | 0.11 | 9.00 | 56.7 | 32.1 | 0 | 2.2 |
|  | 43 | 127.5 | 10.7 | 12.9 | 2.965 | 2.47 | 4.74 | 11.15 | 166.0 | 1527 | 0.15 | 2.03 | 0.34 | 0 | 2.22 | 3.2 | 42.8 | 7.2 | 0 | 46.8 |
|  | 26 | 130.7 | 10.9 | 11.6 | 5.027 | 1.45 | 8.56 | 10.52 | 164.0 | 770 | 0.19 | 5.89 | 0.43 | 0.01 | 2.04 | 2.30 | 68.8 | 5.0 | 0.1 | 23.8 |
|  | 30 | 96.7 | 10.7 | 18.6 | 3.223 | 2.3 | 2.23 | 10.16 | 151.0 | 1038 | 0.07 | 0.22 | 0 | 0.01 | 1.93 | 3.20 | 9.9 | 0 | 0.4 | 86.5 |
|  | 24 | 79.6 | 14.2 | 16.7 | 3.317 | 1.63 | 6.39 | 10.79 | 160.0 | 1084 | 0.42 | 5.45 | 0.38 | 0.01 | 0.13 | 6.60 | 85.3 | 5.9 | 0.2 | 2.0 |
|  | 22 | 105.9 | 6.9 | 15.4 | 4.814 | 1.02 | 7.28 | 10.13 | 147.0 | 782 | 0.14 | 4.67 | 0.42 | 0.01 | 2.04 | 2.0 | 64.1 | 5.8 | 0.1 | 28.0 |
|  | 67 | 210.6 | 7.8 | 23.5 | 3.143 | 1.75 | 9.94 | 10.10 | 149.0 | 1115 | 0.11 | 7.07 | 0 | 0.01 | 2.75 | 1.10 | 71.1 | 0 | 0.1 | 27.7 |
|  | 53 | 203.6 | 7.1 | 10 | 3.842 | 1.33 | 8.05 | 10.13 | 148.0 | 1466 | 0.15 | 5.33 | 1.10 | 0.01 | 1.46 | 1.90 | 66.2 | 13.7 | 0.1 | 18.1 |
|  | 54 | 128.3 | 8.4 | 19.6 | 2.376 | 1.69 | 6.03 | 9.37 | 135.0 | 1540 | 0.12 | 4.99 | 0.27 | 0 | 0.65 | 1.90 | 82.8 | 4.5 | 0 | 10.8 |
| Tregs-treated EAE Group | 18 | 85.1 | 12.7 | 10.3 | 4.728 | 1.93 | 9.63 | 9.68 | 146.0 | 1398 | 0.43 | 6.45 | 1.47 | 0 | 1.28 | 4.4 | 67.0 | 15.3 | 0 | 13.3 |
|  | 37 | 131.9 | 23.3 | 50.8 | 3.565 | 3.07 | 6.24 | 10.61 | 153.0 | 909 | 0.62 | 5.15 | 0.46 | 0.01 | 0 | 9.9 | 82.5 | 7.4 | 0.2 | 0 |
|  | 28 | 130.3 | 9.3 | 11.6 | 4.654 | 1.39 | 7.73 | 10.88 | 158.0 | 1562 | 0.81 | 4.43 | 2.27 | 0.01 | 0.21 | 10.2 | 57.3 | 29.4 | 0.1 | 2.7 |
|  | 18 | 97.6 | 11.8 | 12.2 | 5.422 | 1.75 | 5.67 | 9.76 | 145.0 | 1590 | 0.09 | 4.40 | 0.58 | 0.01 | 0.59 | 1.60 | 77.6 | 10.2 | 0.2 | 10.4 |
|  | 38 | 161.5 | 9.8 | 15.7 | 4.25 | 1.45 | 5.77 | 10.25 | 152.0 | 356 | 0.11 | 4.81 | 0.29 | 0.01 | 0.55 | 1.90 | 83.4 | 5.0 | 0.2 | 9.5 |
|  | 45 | 292.4 | 7.1 | 14.5 | 6.498 | 1.81 | 2.28 | 10.31 | 152.0 | 873 | 0.12 | 1.49 | 0 | 0.01 | 0.66 | 5.30 | 65.4 | 0 | 0.4 | 28.9 |

**Table S2. Kolmogorov-Smirnov test for normality and homogeneity of variance test for body weight of EAE model, WT, Sham-treated EAE and Treg-treatment groups**

| Group | | Kolmogorov-Smirnov test | | |  | Levene's test | | | |
| --- | --- | --- | --- | --- | --- | --- | --- | --- | --- |
|  |  | *D* | *df* | *P* |  | *F* | *df1* | *df2* | *P* |
| Body Weight | Sham | 0.046 | 190 | 0.200 |  | 2.742 | 1 | 1178 | 0.098 |
|  | EAE | 0.033 | 990 | 0.012 |  |  |  |  |  |
| Body Weight | WT | 0.085 | 784 | <0.001 |  | 12.327 | 2 | 2685 | <0.001 |
|  | Sham-treated EAE | 0.024 | 1120 | 0.122 |  |  |  |  |  |
|  | Treg-treated EAE | 0.062 | 784 | <0.001 |  |  |  |  |  |

**Table S3. Generalized linear mixed model (GLMM) was used for body weight and clinical scores of EAE model**

| Source | |  | Fixed effect | | | |
| --- | --- | --- | --- | --- | --- | --- |
|  |  | *F* | | *df1* | *df2* | *P* |
| Body Weight | Calibration model | 9.000 | | 37 | 1142 | <0.001 |
|  | EAE | 19.176 | | 1 | 1142 | <0.001 |
|  | Time | 4.874 | | 18 | 1142 | <0.001 |
|  | EAE*Time | 5.355 | | 18 | 1142 | 0.000 |
| Clinical Scores | Calibration model | 21.378 | | 37 | 1143 | <0.001 |
|  | EAE | 29.400 | | 1 | 1143 | <0.001 |
|  | Time | 6.747 | | 18 | 1143 | <0.001 |
|  | EAE*Time | 6.747 | | 18 | 1143 | <0.001 |

**Table S4. Pairwise comparisons in GLMM have been done for EAE model**

| Days post-immunization | Cinical scores | | | |  | Body Weight (g) | | | |
| --- | --- | --- | --- | --- | --- | --- | --- | --- | --- |
|  | *diff. (95% CI)* | *SE* | *t* | *P* |  | *diff. (95% CI)* | *SE* | *t* | *P* |
| 0 | -6.217E-15 (-0.438, 0.438) | 0.223 | -2.784E-14 | >0.999 |  | -0.483(-1.759, 0.793) | 0.650 | -0.742 | 0.458 |
| 1 | -1.199E-14 (-0.438, 0.438) | 0.223 | -5.369E-14 | >0.999 |  | -0.366(-1.643, 0.910) | 0.650 | -0.563 | 0.573 |
| 2 | -9.770E-15 (-0.438, 0.438) | 0.223 | -4.374E-14 | >0.999 |  | -0.145(-1.421, 1.132) | 0.650 | -0.222 | 0.824 |
| 3 | -9.326E-15 (-0.438, 0.438) | 0.223 | -4.176E-14 | >0.999 |  | 0.195(-1.081,1.471) | 0.650 | 0.299 | 0.765 |
| 4 | -7.550E-15 (-0.438, 0.438) | 0.223 | -3.380E-14 | >0.999 |  | 0.579(-0.697,1.855) | 0.650 | 0.890 | 0.373 |
| 5 | -5.329E-15 (-0.438, 0.438) | 0.223 | -2.386E-14 | >0.999 |  | 0.849(-0.427,2.125) | 0.650 | 1.305 | 0.192 |
| 6 | -4.885E-15 (-0.438, 0.438) | 0.223 | -2.187E-14 | >0.999 |  | 0.766(-0.510,2.042) | 0.650 | 1.178 | 0.239 |
| 7 | -2.220E-15 (-0.438, 0.438) | 0.223 | -9.942E-15 | >0.999 |  | 1.181(-0.096,2.457) | 0.650 | 1.815 | 0.070 |
| 8 | -4.441E-16 (-0.438, 0.438) | 0.223 | -1.988E-15 | >0.999 |  | 0.948(-0.328, 2.225) | 0.650 | 1.458 | 0.145 |
| 9 | -0.057 (-0.495, 0.382) | 0.223 | -0.253 | 0.800 |  | 1.232(-0.044, 2.508) | 0.650 | 1.894 | 0.058 |
| 10 | -0.170 (-0.608, 0.268) | 0.223 | -0.760 | 0.447 |  | 1.134(-0.142,2.410) | 0.650 | 1.743 | 0.082 |
| 11 | -0.443 (-0.882, -0.005) | 0.223 | -1.985 | 0.047 |  | 1.737(0.461,3.014) | 0.651 | 2.670 | 0.008 |
| 12 | -0.735 (-1.173, -0.296) | 0.223 | -3.288 | 0.001 |  | 2.677(1. 400, 3.954) | 0.651 | 4.113 | <0.001 |
| 13 | -1.215 (-1.654, -0.776) | 0.224 | -5.433 | <0.001 |  | 3.553(2.275,4.830) | 0.651 | 5.457 | <0.001 |
| 14 | -1.878 (-2.317, -1.439) | 0.224 | -8.390 | <0.001 |  | 4.617(3.339, 5.895) | 0.651 | 7.089 | <0.001 |
| 15 | -2.172 (-2.611, -1.733) | 0.224 | -9.702 | <0.001 |  | 5.346(4.068,6.624) | 0.651 | 8.206 | <0.001 |
| 16 | -2.450 (-2.890. -2.011) | 0.224 | -10.943 | <0.001 |  | 5.741(4.463,7.020) | 0.652 | 8.810 | <0.001 |
| 17 | -2.538 (-2.978, -2.098) | 0.224 | -11.325 | <0.001 |  | 5.676(4.397, 6.956) | 0.652 | 8.706 | <0.001 |
| 18 | -2.591 (-3.031, -2.151) | 0.224 | -11.552 | <0.001 |  | 6.259(4.979,7.539) | 0.652 | 9.593 | <0.001 |

**Table S5. Generalized linear mixed model (GLMM) has been performed for body weight and clinical scores of WT, Sham-treated EAE and Treg-treated EAE groups**

| **Source** | | **Fixed effect** | | | |  | **Pairwise comparison** | | | |
| --- | --- | --- | --- | --- | --- | --- | --- | --- | --- | --- |
|  |  | *F* | *df1* | *df2* | *P* |  |  | WT-sham-treated EAE | WT-Treg-treated EAE | sham-treated EAE- Treg-treated EAE |
| **Body Weight** | Calibration model | 3.859 | 167 | 2520 | <0.001 |  | *diff (95%CI)* | 1.628 (0.941, 2.315) | 1.447 (0.702, 2.193) | -0.181 (-0.868, 0.507) |
|  | Treatment | 11.959 | 2 | 2520 | <0.001 |  | *SE* | 0.351 | 0.380 | 0.351 |
|  | Time | 6.957 | 55 | 2520 | <0.001 |  | *t* | 4.644 | 3.806 | -0.516 |
|  | Treatment*Time | 2.015 | 110 | 2520 | <0.001 |  | *P* | <0.001 | <0.001 | 0.606 |
| **Clinical Scores** | Calibration model | 47.153 | 5 | 2682 | <0.001 |  | *diff (95%CI)* | -2.822 (-3.307, -2.338) | -1.899 (-2.424, -1.373) | 0.923 (0.439, 1.408) |
|  | Treatment | 72.023 | 1 | 2682 | <0.001 |  | *SE* | 0.247 | 0.268 | 0.247 |
|  | Time | 1.427 | 2 | 2682 | 0.240 |  | *t* | -11.425 | -7.087 | 3.738 |
|  | Treatment*Time | 21.063 | 2 | 2682 | <0.001 |  | *P* | ＜0.001 | <0.001 | <0.001 |

**Table S6. Two-way ANOVA for repeated measures with Tukey's multiple comparisons has been performed in WT, sham-treated EAE and Treg-treated EAE groups**

| Days post-immunization (d) | Tukey’s multiple comparisons | Mean clinical scores | |  | Body Weight (g) | |
| --- | --- | --- | --- | --- | --- | --- |
|  |  | *mean diff. (95% CI)* | *P* |  | *mean diff. (95% CI)* | *P* |
| 0 | WT vs. Sham-treated EAE | 0 |  |  | -0.630 (-1.525, 0.265) | 0.204 |
|  | WT vs. Treg-treated EAE | 0 |  |  | -0.364 (-1.226, 0.498) | 0.542 |
|  | Sham-treated EAE vs. Treg-treated EAE | 0 |  |  | 0.265 (-0.336, 0.867) | 0.530 |
| 1 | WT vs. Sham-treated EAE | 0 |  |  | -0.859 (-1.851, 0.133) | 0.099 |
|  | WT vs. Treg-treated EAE | 0 |  |  | -0.626 (-1.578, 0.326) | 0.244 |
|  | Sham-treated EAE vs. Treg-treated EAE | 0 |  |  | 0.233 (-0.468, 0.935) | 0.695 |
| 2 | WT vs. Sham-treated EAE | 0 |  |  | -0.564 (-1.694, 0.566) | 0.443 |
|  | WT vs. Treg-treated EAE | 0 |  |  | -0.266 (-1.331, 0.798) | 0.807 |
|  | Sham-treated EAE vs. Treg-treated EAE | 0 |  |  | 0.298 (-0.625, 1.221) | 0.710 |
| 3 | WT vs. Sham-treated EAE | 0 |  |  | -0.253 (-1.199, 0.694) | 0.788 |
|  | WT vs. Treg-treated EAE | 0 |  |  | -0.106 (-1.090, 0.879) | 0.962 |
|  | Sham-treated EAE vs. Treg-treated EAE | 0 |  |  | 0.147 (-0.749, 1.043) | 0.914 |
| 4 | WT vs. Sham-treated EAE | 0 |  |  | -0.030 (-1.051, 0.991) | 0.997 |
|  | WT vs. Treg-treated EAE | 0 |  |  | 0.217 (-0.758, 1.193) | 0.844 |
|  | Sham-treated EAE vs. Treg-treated EAE | 0 |  |  | 0.247 (-0.583, 1.077) | 0.746 |
| 5 | WT vs. Sham-treated EAE | 0 |  |  | -0.311 (-1.281, 0.659) | 0.708 |
|  | WT vs. Treg-treated EAE | 0 |  |  | -0.169 (-1.088, 0.751) | 0.889 |
|  | Sham-treated EAE vs. Treg-treated EAE | 0 |  |  | 0.142 (-0.571, 0.855) | 0.877 |
| 6 | WT vs. Sham-treated EAE | 0 |  |  | -0.240 (-1.255, 0.776) | 0.828 |
|  | WT vs. Treg-treated EAE | 0 |  |  | -0.065 (-0.996, 0.866) | 0.983 |
|  | Sham-treated EAE vs. Treg-treated EAE | 0 |  |  | 0.175 (-0.531, 0.881) | 0.816 |
| 7 | WT vs. Sham-treated EAE | 0 |  |  | -0.295 (-1.430, 0.840) | 0.794 |
|  | WT vs. Treg-treated EAE | 0 |  |  | -0.261 (-1.300, 0.779) | 0.795 |
|  | Sham-treated EAE vs. Treg-treated EAE | 0 |  |  | 0.034 (-0.633, 0.701) | 0.991 |
| 8 | WT vs. Sham-treated EAE | 0 |  |  | -0.338 (-1.545, 0.869) | 0.768 |
|  | WT vs. Treg-treated EAE | 0 |  |  | -0.634 (-1.785, 0.517) | 0.366 |
|  | Sham-treated EAE vs. Treg-treated EAE | 0 |  |  | -0.296 (-1.230, 0.637) | 0.718 |
| 9 | WT vs. Sham-treated EAE | 0 |  |  | -0.336 (-1.564, 0.893) | 0.773 |
|  | WT vs. Treg-treated EAE | 0 |  |  | -0.341 (-1.547, 0.866) | 0.757 |
|  | Sham-treated EAE vs. Treg-treated EAE | 0 |  |  | -0.005 (-0.806, 0.795) | 1.000 |
| 10 | WT vs. Sham-treated EAE | -0.050 (-0.177, 0.077) | 0.586 |  | -0.745 (-1.899, 0.409) | 0.262 |
|  | WT vs. Treg-treated EAE | 0 |  |  | -0.790 (-1.871, 0.291) | 0.181 |
|  | Sham-treated EAE vs. Treg-treated EAE | 0.050 (-0.077, 0.177) | 0.586 |  | -0.045 (-0.932, 0.842) | 0.992 |
| 11 | WT vs. Sham-treated EAE | -0.100 (-0.298, 0.098) | 0.420 |  | -0.521 (-1.644, 0.602) | 0.495 |
|  | WT vs. Treg-treated EAE | -0.107 (-0.311, 0.097) | 0.377 |  | -0.439 (-1.594, 0.716) | 0.617 |
|  | Sham-treated EAE vs. Treg-treated EAE | -0.007 (-0.277, 0.263) | 0.998 |  | 0.082 (-1.010, 1.174) | 0.981 |
| 12 | WT vs. Sham-treated EAE | -0.350 (-0.696, -0.004) | 0.047 |  | -0.219 (-1.517, 1.079) | 0.909 |
|  | WT vs. Treg-treated EAE | -0.500 (-1.036, 0.036) | 0.069 |  | -0.199 (-1.386, 0.989) | 0.908 |
|  | Sham-treated EAE vs. Treg-treated EAE | -0.150 (-0.761, 0.461) | 0.814 |  | 0.020 (-1.461,1.502) | 0.999 |
| 13 | WT vs. Sham-treated EAE | -0.850 (-1.338, -0.362) | 0.001 |  | 1.154 (-0.295, 2.602) | 0.139 |
|  | WT vs. Treg-treated EAE | -0.607 (-1.213, -0.001) | 0.050 |  | 0.660 (-0.452, 1.772) | 0.319 |
|  | Sham-treated EAE vs. Treg-treated EAE | 0.243 (-0.498, 0.984) | 0.699 |  | -0.494 (-1.919, 0.931) | 0.673 |
| 14 | WT vs. Sham-treated EAE | -1.400 (-2.049, -0.751) | <0.001 |  | 1.695 (0.230, 3.160) | 0.021 |
|  | WT vs. Treg-treated EAE | -1.000 (-1.875, -0.125) | 0.025 |  | 1.119 (-0.222, 2.460) | 0.114 |
|  | Sham-treated EAE vs. Treg-treated EAE | 0.400 (-0.639, 1.439) | 0.611 |  | -0.576 (-2.254, 1.102) | 0.679 |
| 15 | WT vs. Sham-treated EAE | -1.875 (-2.634, -1.116) | <0.001 |  | 1.788 (0.265, 3.311) | 0.019 |
|  | WT vs. Treg-treated EAE | -1.214 (-2.160, -0.268) | 0.013 |  | 1.016 (-0.536, 2.567) | 0.251 |
|  | Sham-treated EAE vs. Treg-treated EAE | 0.661 (-0.493, 1.815) | 0.346 |  | -0.772 (-2.509, 0.966) | 0.525 |
| 16 | WT vs. Sham-treated EAE | -2.375 (-3.039, -1.711) | <0.001 |  | 2.456 (0.925, 3.988) | 0.001 |
|  | WT vs. Treg-treated EAE | -1.429 (-2.416, -0.442) | 0.006 |  | 1.643 (-0.140, 3.426) | 0.075 |
|  | Sham-treated EAE vs. Treg-treated EAE | 0.946 (-0.190, 2.083) | 0.116 |  | -0.814 (-2.723, 1.096) | 0.550 |
| 17 | WT vs. Sham-treated EAE | -2.525 (-3.153, -1.897) | <0.001 |  | 2.390 (0.934, 3.847) | 0.001 |
|  | WT vs. Treg-treated EAE | -1.607 (-2.587, -0.627) | 0.002 |  | 1.573 (0.287, 2.859) | 0.014 |
|  | Sham-treated EAE vs. Treg-treated EAE | 0.918 (-0.197, 2.032) | 0.120 |  | -0.818 (-2.321, 0.686) | 0.386 |
| 18 | WT vs. Sham-treated EAE | -2.775 (-3.308, -2.242) | <0.001 |  | 2.437 (1.183, 3.691) | 0.000 |
|  | WT vs. Treg-treated EAE | -1.679 (-2.720, -0.637) | 0.003 |  | 1.804 (0.654, 2.954) | 0.002 |
|  | Sham-treated EAE vs. Treg-treated EAE | 1.096 (-0.032, 2.225) | 0.058 |  | -0.633 (-1.945, 0.680) | 0.471 |
| 19 | WT vs. Sham-treated EAE | -2.700 (-3.136, -2.264) | <0.001 |  | 2.313 (1.236, 3.390) | <0.001 |
|  | WT vs. Treg-treated EAE | -1.679 (-2.643, -0.714) | 0.001 |  | 2.276 (1.033, 3.519) | 0.001 |
|  | Sham-treated EAE vs. Treg-treated EAE | 1.021 (-0.005, 2.048) | 0.051 |  | -0.037 (-1.444, 1.369) | 0.998 |
| 20 | WT vs. Sham-treated EAE | -2.650 (-3.042, -2.258) | <0.001 |  | 2.398 (1.247, 3.549) | <0.001 |
|  | WT vs. Treg-treated EAE | -1.643 (-2.576, -0.710) | 0.001 |  | 2.324 (0.784, 3.863) | 0.003 |
|  | Sham-treated EAE vs. Treg-treated EAE | 1.007 (0.023, 1.991) | 0.044 |  | -0.075 (-1.665, 1.516) | 0.993 |
| 21 | WT vs. Sham-treated EAE | -2.550 (-2.940, -2.160) | <0.001 |  | 2.260 (0.962, 3.559) | 0.001 |
|  | WT vs. Treg-treated EAE | -1.464 (-2.300, -0.629) | 0.001 |  | 2.114 (0.414, 3.813) | 0.013 |
|  | Sham-treated EAE vs. Treg-treated EAE | 1.086 (0.193, 1.979) | 0.016 |  | -0.147 (-1.844, 1.550) | 0.975 |
| 22 | WT vs. Sham-treated EAE | -2.475 (-2.882, -2.068) | <0.001 |  | 2.178 (1.076, 3.280) | <0.001 |
|  | WT vs. Treg-treated EAE | -1.464 (-2.277, -0.652) | 0.001 |  | 2.055 (0.647, 3.463) | 0.004 |
|  | Sham-treated EAE vs. Treg-treated EAE | 1.011 (0.133, 1.888) | 0.022 |  | -0.123 (-1.620, 1.374) | 0.977 |
| 23 | WT vs. Sham-treated EAE | -2.425 (-2.830, -2.020) | <0.001 |  | 2.350 (1.359, 3.340) | <0.001 |
|  | WT vs. Treg-treated EAE | -1.500 (-2.307, -0.693) | 0.001 |  | 1.853 (0.246, 3.460) | 0.022 |
|  | Sham-treated EAE vs. Treg-treated EAE | 0.925 (0.053, 1.797) | 0.036 |  | -0.497 (-2.101, 1.108) | 0.717 |
| 24 | WT vs. Sham-treated EAE | -2.400 (-2.797, -2.003) | <0.001 |  | 2.314 (1.262, 3.366) | <0.001 |
|  | WT vs. Treg-treated EAE | -1.464 (-2.241, -0.688) | 0.001 |  | 1.841 (0.448, 3.235) | 0.009 |
|  | Sham-treated EAE vs. Treg-treated EAE | 0.936 (0.094, 1.777) | 0.028 |  | -0.473 (-1.911, 0.966) | 0.695 |
| 25 | WT vs. Sham-treated EAE | -2.300 (-2.673, -1.927) | <0.001 |  | 2.575 (1.534, 3.616) | <0.001 |
|  | WT vs. Treg-treated EAE | -1.429 (-2.119, -0.739) | 0.000 |  | 2.180 (0.787, 3.573) | 0.002 |
|  | Sham-treated EAE vs. Treg-treated EAE | 0.871 (0.116, 1.627) | 0.022 |  | -0.395 (-1.784, 0.995) | 0.759 |
| 26 | WT vs. Sham-treated EAE | -2.225 (-2.576, -1.874) | <0.001 |  | 2.481 (1.212, 3.751) | <0.001 |
|  | WT vs. Treg-treated EAE | -1.643 (-2.381, -0.905) | 0.000 |  | 2.442 (0.9433, 3.941) | 0.001 |
|  | Sham-treated EAE vs. Treg-treated EAE | 0.582 (-0.209, 1.373) | 0.175 |  | -0.039 (-1.500, 1.422) | 0.998 |
| 27 | WT vs. Sham-treated EAE | -2.225 (-2.599, -1.851) | <0.001 |  | 2.629 (1.430, 3.828) | <0.001 |
|  | WT vs. Treg-treated EAE | -1.929 (-2.672, -1.185) | <0.001 |  | 2.738 (1.486, 3.990) | <0.0001 |
|  | Sham-treated EAE vs. Treg-treated EAE | 0.296 (-0.507, 1.100) | 0.626 |  | 0.109 (-1.217, 1.435) | 0.978 |
| 28 | WT vs. Sham-treated EAE | -2.325 (-2.648, -2.002) | <0.001 |  | 2.489 (1.329, 3.648) | <0.001 |
|  | WT vs. Treg-treated EAE | -2.143 (-2.650, -1.636) | <0.001 |  | 3.168 (1.893, 4.443) | <0.001 |
|  | Sham-treated EAE vs. Treg-treated EAE | 0.182 (-0.393, 0.758) | 0.712 |  | 0.679 (-0.648, 2.006) | 0.426 |
| 29 | WT vs. Sham-treated EAE | -2.375 (-2.693, -2.057) | <0.001 |  | 2.537 (1.234, 3.840) | 0.000 |
|  | WT vs. Treg-treated EAE | -2.143 (-2.631, -1.655) | <0.001 |  | 3.144 (1.620, 4.668) | <0.001 |
|  | Sham-treated EAE vs. Treg-treated EAE | 0.232 (-0.325, 0.789) | 0.559 |  | 0.607 (-0.723, 1.936) | 0.501 |
| 30 | WT vs. Sham-treated EAE | -2.400 (-2.727, -2.073) | <0.001 |  | 3.244 (1.922, 4.566) | <0.001 |
|  | WT vs. Treg-treated EAE | -2.214 (-2.646, -1.783) | <0.001 |  | 3.206 (1.731,4.680) | <0.001 |
|  | Sham-treated EAE vs. Treg-treated EAE | 0.186 (-0.330, 0.701) | 0.649 |  | -0.038 (-1.524, 1.448) | 0.998 |
| 31 | WT vs. Sham-treated EAE | -2.500 (-2.776, -2.224) | <0.001 |  | 3.140 (1.900, 4.381) | <0.001 |
|  | WT vs. Treg-treated EAE | -1.964 (-2.433, -1.496) | <0.001 |  | 3.277 (1.765, 4.790) | <0.001 |
|  | Sham-treated EAE vs. Treg-treated EAE | 0.536 (0.013, 1.058) | 0.044 |  | 0.137 (-1.363, 1.637) | 0.972 |
| 32 | WT vs. Sham-treated EAE | -2.450 (-2.740, -2.160) | <0.001 |  | 2.975 (1.582, 4.368) | <0.001 |
|  | WT vs. Treg-treated EAE | -1.893 (-2.395, -1.390) | <0.001 |  | 3.022 (1.559, 4.485) | <0.001 |
|  | Sham-treated EAE vs. Treg-treated EAE | 0.557 (-0.000, 1.114) | 0.050 |  | 0.047 (-1.611, 1.706) | 0.997 |
| 33 | WT vs. Sham-treated EAE | -2.525 (-2.863, -2.187) | <0.001 |  | 2.401 (1.335, 3.467) | <0.001 |
|  | WT vs. Treg-treated EAE | -1.893 (-2.395, -1.390) | <0.001 |  | 2.072 (0.473, 3.672) | 0.010 |
|  | Sham-treated EAE vs. Treg-treated EAE | 0.632 (0.053, 1.211) | 0.030 |  | -0.329 (-1.907, 1.250) | 0.860 |
| 34 | WT vs. Sham-treated EAE | -2.475 (-2.849, -2.101) | <0.001 |  | 2.446 (1.263, 3.628) | <0.001 |
|  | WT vs. Treg-treated EAE | -1.893 (-2.395, -1.390) | <0.001 |  | 2.489 (1.041, 3.936) | 0.001 |
|  | Sham-treated EAE vs. Treg-treated EAE | 0.582 (-0.015, 1.179) | 0.057 |  | 0.043 (-1.311, 1.397) | 0.997 |
| 35 | WT vs. Sham-treated EAE | -2.425 (-2.748, -2.102) | <0.001 |  | 2.548 (1.395, 3.702) | <0.001 |
|  | WT vs. Treg-treated EAE | -1.857 (-2.383, -1.331) | <0.001 |  | 2.369 (0.845, 3.893) | 0.002 |
|  | Sham-treated EAE vs. Treg-treated EAE | 0.568 (-0.023, 1.159) | 0.061 |  | -0.179 (-1.700, 1.342) | 0.954 |
| 36 | WT vs. Sham-treated EAE | -2.375 (-2.730, -2.020) | <0.001 |  | 2.626 (1.365, 3.887) | <0.001 |
|  | WT vs. Treg-treated EAE | -1.750 (-2.299, -1.201) | <0.001 |  | 2.574 (1.041, 4.107) | 0.001 |
|  | Sham-treated EAE vs. Treg-treated EAE | 0.625 (-0.001, 1.251) | 0.050 |  | -0.052 (-1.549, 1.446) | 0.996 |
| 37 | WT vs. Sham-treated EAE | -2.300 (-2.650, -1.950) | <0.001 |  | 1.902 (0.876, 2.927) | <0.001 |
|  | WT vs. Treg-treated EAE | -1.714 (-2.280, -1.148) | <0.001 |  | 2.326 (0.701, 3.950) | 0.005 |
|  | Sham-treated EAE vs. Treg-treated EAE | 0.586 (-0.052, 1.223) | 0.076 |  | 0.424 (-1.227, 2.075) | 0.795 |
| 38 | WT vs. Sham-treated EAE | -2.175 (-2.590, -1.760) | <0.001 |  | 2.170 (1.018, 3.321) | <0.001 |
|  | WT vs. Treg-treated EAE | -1.679 (-2.291, -1.066) | <0.001 |  | 2.504 (1.067, 3.942) | 0.001 |
|  | Sham-treated EAE vs. Treg-treated EAE | 0.496 (-0.211, 1.204) | 0.207 |  | 0.335 (-1.080, 1.749) | 0.827 |
| 39 | WT vs. Sham-treated EAE | -2.300 (-2.782, -1.818) | <0.001 |  | 1.791 (0.774, 2.808) | <0.001 |
|  | WT vs. Treg-treated EAE | -1.679 (-2.406, -0.951) | <0.001 |  | 2.446 (0.913, 3.978) | 0.002 |
|  | Sham-treated EAE vs. Treg-treated EAE | 0.621 (-0.213, 1.456) | 0.172 |  | 0.655 (-0.838, 2.147) | 0.519 |
| 40 | WT vs. Sham-treated EAE | -2.325 (-2.708, -1.942) | <0.001 |  | 1.745 (0.610, 2.880) | 0.002 |
|  | WT vs. Treg-treated EAE | -1.679 (-2.406, -0.951) | 0.000 |  | 2.336 (0.586, 4.087) | 0.008 |
|  | Sham-treated EAE vs. Treg-treated EAE | 0.646 (-0.146, 1.439) | 0.123 |  | 0.592 (-0.999, 2.182) | 0.614 |
| 41 | WT vs. Sham-treated EAE | -2.425 (-2.819, -2.031) | <0.001 |  | 2.241 (1.109, 3.373) | <0.001 |
|  | WT vs. Treg-treated EAE | -2.036 (-2.692, -1.380) | <0.001 |  | 2.664 (0.761, 4.568) | 0.006 |
|  | Sham-treated EAE vs. Treg-treated EAE | 0.389 (-0.345, 1.123) | 0.394 |  | 0.423 (-1.452, 2.298) | 0.835 |
| 42 | WT vs. Sham-treated EAE | -2.400 (-2.752, -2.048) | <0.001 |  | 1.944 (0.794, 3.094) | 0.001 |
|  | WT vs. Treg-treated EAE | -1.929 (-2.497, -1.360) | <0.001 |  | 2.624 (1.162, 4.085) | <0.001 |
|  | Sham-treated EAE vs. Treg-treated EAE | 0.471 (-0.169, 1.112) | 0.178 |  | 0.679 (-0.659, 2.018) | 0.424 |
| 43 | WT vs. Sham-treated EAE | -2.350 (-2.720, -1.980) | <0.001 |  | 1.926 (0.838, 3.013) | 0.001 |
|  | WT vs. Treg-treated EAE | -1.929 (-2.497, -1.360) | <0.001 |  | 2.282 (0.768, 3.796) | 0.003 |
|  | Sham-treated EAE vs. Treg-treated EAE | 0.421 (-0.227, 1.070) | 0.256 |  | 0.357 (-1.044, 1.757) | 0.798 |
| 44 | WT vs. Sham-treated EAE | -2.350 (-2.708, -1.992) | <0.001 |  | 2.423 (1.206, 3.639) | <0.001 |
|  | WT vs. Treg-treated EAE | -1.821 (-2.385, -1.257) | <0.001 |  | 2.151 (0.614, 3.687) | 0.005 |
|  | Sham-treated EAE vs. Treg-treated EAE | 0.529 (-0.111, 1.168) | 0.119 |  | -0.272 (-1.761, 1.217) | 0.892 |
| 45 | WT vs. Sham-treated EAE | -2.375 (-2.730, -2.020) | <0.001 |  | 2.083 (0.983, 3.182) | <0.001 |
|  | WT vs. Treg-treated EAE | -1.679 (-2.208, -1.150) | <0.001 |  | 1.798 (0.214, 3.382) | 0.024 |
|  | Sham-treated EAE vs. Treg-treated EAE | 0.696 (0.087, 1.305) | 0.023 |  | -0.285 (-1.885, 1.316) | 0.896 |
| 46 | WT vs. Sham-treated EAE | -2.250 (-2.601, -1.899) | <0.001 |  | 2.310 (1.110, 3.509) | <0.001 |
|  | WT vs. Treg-treated EAE | -1.714 (-2.263, -1.166) | <0.001 |  | 1.584 (0.122, 3.047) | 0.032 |
|  | Sham-treated EAE vs. Treg-treated EAE | 0.536 (-0.088, 1.159) | 0.102 |  | -0.725 (-2.237, 0.787) | 0.469 |
| 47 | WT vs. Sham-treated EAE | -2.375 (-2.730, -2.020) | <0.001 |  | 1.737 (0.411, 3.062) | 0.008 |
|  | WT vs. Treg-treated EAE | -1.679 (-2.243, -1.115) | <0.001 |  | 1.375 (-0.189, 2.939) | 0.093 |
|  | Sham-treated EAE vs. Treg-treated EAE | 0.696 (0.058, 1.335) | 0.031 |  | -0.362 (-1.881, 1.158) | 0.826 |
| 48 | WT vs. Sham-treated EAE | -2.450 (-2.817, -2.083) | <0.001 |  | 1.783 (0.338, 3.228) | 0.013 |
|  | WT vs. Treg-treated EAE | -1.643 (-2.204, -1.082) | <0.001 |  | 1.182 (-0.522, 2.886) | 0.215 |
|  | Sham-treated EAE vs. Treg-treated EAE | 0.807 (0.166, 1.448) | 0.012 |  | -0.601 (-2.140, 0.939) | 0.601 |
| 49 | WT vs. Sham-treated EAE | -2.275 (-2.573, -1.977) | <0.001 |  | 2.204 (0.732, 3.675) | 0.003 |
|  | WT vs. Treg-treated EAE | -1.571 (-2.156, -0.987) | <0.001 |  | 1.245 (-0.390, 2.880) | 0.161 |
|  | Sham-treated EAE vs. Treg-treated EAE | 0.704 (0.070, 1.337) | 0.028 |  | -0.959 (-2.335, 0.417) | 0.213 |
| 50 | WT vs. Sham-treated EAE | -2.375 (-2.742, -2.008) | <0.001 |  | 1.906 (0.375, 3.436) | 0.013 |
|  | WT vs. Treg-treated EAE | -1.500 (-2.054, -0.946) | <0.001 |  | 0.933 (-0.765, 2.630) | 0.372 |
|  | Sham-treated EAE vs. Treg-treated EAE | 0.875 (0.240, 1.510) | 0.006 |  | -0.973 (-2.334, 0.388) | 0.197 |
| 51 | WT vs. Sham-treated EAE | -2.375 (-2.679, -2.071) | <0.001 |  | 2.016 (0.468, 3.564) | 0.009 |
|  | WT vs. Treg-treated EAE | -1.500 (-2.071, -0.929) | <0.001 |  | 1.178 (-0.484, 2.840) | 0.202 |
|  | Sham-treated EAE vs. Treg-treated EAE | 0.875 (0.252, 1.498) | 0.005 |  | -0.838 (-2.201, 0.525) | 0.296 |
| 52 | WT vs. Sham-treated EAE | -2.325 (-2.590, -2.060) | <0.001 |  | 1.758 (0.181, 3.334) | 0.027 |
|  | WT vs. Treg-treated EAE | -1.393 (-1.983, -0.803) | <0.001 |  | 0.866 (-0.820, 2.551) | 0.420 |
|  | Sham-treated EAE vs. Treg-treated EAE | 0.932 (0.305, 1.560) | 0.004 |  | -0.892 (-2.277, 0.493) | 0.265 |
| 53 | WT vs. Sham-treated EAE | -2.425 (-2.785, -2.065) | <0.001 |  | 2.183 (0.473, 3.894) | 0.011 |
|  | WT vs. Treg-treated EAE | -1.286 (-1.945, -0.626) | 0.001 |  | 0.869 (-0.987, 2.726) | 0.485 |
|  | Sham-treated EAE vs. Treg-treated EAE | 1.139 (0.416, 1.863) | 0.002 |  | -1.314 (-2.891, 0.263) | 0.116 |
| 54 | WT vs. Sham-treated EAE | -2.375 (-2.718, -2.032) | <0.001 |  | 2.213 (0.538, 3.888) | 0.008 |
|  | WT vs. Treg-treated EAE | -1.464 (-2.105, -0.823) | <0.001 |  | 0.735 (-1.144, 2.614) | 0.601 |
|  | Sham-treated EAE vs. Treg-treated EAE | 0.911 (0.210, 1.611) | 0.010 |  | -1.478 (-3.139, 0.183) | 0.088 |
| 55 | WT vs. Sham-treated EAE | -2.300 (-2.625, -1.975) | <0.001 |  | 2.342 (0.866, 3.818) | 0.001 |
|  | WT vs. Treg-treated EAE | -1.429 (-2.090, -0.767) | <0.001 |  | 1.026 (-0.666, 2.717) | 0.304 |
|  | Sham-treated EAE vs. Treg-treated EAE | 0.871 (0.159, 1.584) | 0.015 |  | 1.317 (-0.243, 2.876) | 0.110 |

**Table S7. Normal distribution testing using Kolmogorov-Smirnov test and homogeneity of variance for demyelination (%)**

| Group | | Kolmogorov-Smirnov test | | |  | Levene's test | | | |
| --- | --- | --- | --- | --- | --- | --- | --- | --- | --- |
|  |  | *D* | *df* | *P* |  | *F* | *df1* | *df2* | *P* |
| Demyelination (%) | WT | - | 21 | - |  | 17.609 | 2 | 69 | <0.001 |
|  | Sham-treated EAE | 0.103 | 30 | 0.200 |  |  |  |  |  |
|  | Treg-treated EAE | 0.170 | 21 | 0.114 |  |  |  |  |  |

**Table S8. Kruskal-Wallis test for inflammatory scores and demyelination (%)**

|  | Group | Description | | |  | Multiple Comparison | | | |
| --- | --- | --- | --- | --- | --- | --- | --- | --- | --- |
|  |  | *N* | *Median* | *IQR* |  | *Groups* | *H* | *SE* | *P^a^* |
| Inflammatory Scores | WT | 105 | 0 | 0 |  | WT vs Sham-treated EAE | -200.600 | 12.655 | <0.001 |
|  | Sham-treated EAE | 150 | 4 | 1 |  | WT vs Treg-treated EAE | -116.286 | 13.726 | <0.001 |
|  | Treg-treated EAE | 105 | 3 | 2 |  | Treg-treated EAE vs Sham-treated EAE | 84.314 | 12.655 | <0.001 |
|  | *H* | 251.296 | | |  |  |  |  |  |
|  | *df* | 2 | | |  |  |  |  |  |
|  | *P* | <0.001 | | |  |  |  |  |  |
| Demyelination (%) | WT | 21 | 0 | 0 |  | WT vs Sham-treated EAE | -44.000 | 5.880 | <0.001 |
|  | Sham-treated EAE | 30 | 21.04 | 13.42 |  | WT vs Treg-treated EAE | -24.571 | 6.378 | <0.001 |
|  | Treg-treated EAE | 21 | 9.22 | 10.13 |  | Treg-treated EAE vs Sham-treated EAE | 19.429 | 5.880 | 0.003 |
|  | *H* | 56.048 | | |  |  |  |  |  |
|  | *df* | 2 | | |  |  |  |  |  |
|  | *P* | <0.001 | | |  |  |  |  |  |

a: Multiple comparisons have been adjusted by Bonferroni.

**Table S9. Kolmogorov-Smirnov test for normality and homogeneity of variance test for immunofluorescence analysis**

| Group | | Kolmogorov-Smirnov test | | |  | Levene's test | | | |
| --- | --- | --- | --- | --- | --- | --- | --- | --- | --- |
|  |  | *D* | *df* | *P* |  | *F* | *df1* | *df2* | *P* |
| DAPI^+^CCN3^+^ cells / /mm^2^ | WT | 0.199 | 10 | 0.200 |  | 0.155 | 2 | 27 | 0.857 |
|  | Sham-treated EAE | 0.140 | 10 | 0.200 |  |  |  |  |  |
|  | Treg-treated EAE | 0.185 | 10 | 0.200 |  |  |  |  |  |
| CCN3^+^Olig2^+^ cells / mm^2^ | WT | 0.167 | 10 | 0.200 |  | 0.074 | 2 | 27 | 0.929 |
|  | Sham-treated EAE | 0.173 | 10 | 0.200 |  |  |  |  |  |
|  | Treg-treated EAE | 0.198 | 10 | 0.200 |  |  |  |  |  |
| CCN3^+^Olig2^+^/Olig2^+^ cells (%) | WT | 0.180 | 10 | 0.200 |  | 0.242 | 2 | 27 | 0.787 |
|  | Sham-treated EAE | 0.145 | 10 | 0.200 |  |  |  |  |  |
|  | Treg-treated EAE | 0.169 | 10 | 0.200 |  |  |  |  |  |

**Table S10. One-way ANOVA analysis for immunofluorescence analysis**

|  | Group | Description | |  | Multiple Comparison | | | |
| --- | --- | --- | --- | --- | --- | --- | --- | --- |
|  |  | *N* | *Mean ± SD* |  | *Groups* | *Mean Range* | *SE* | *P* |
| DAPI^+^CCN3^+^ cells/mm^2^ | WT | 10 | 432.40 ± 90.709 |  | WT vs Sham-treated EAE | 70.700 | 46.951 | 0.431 |
|  | Sham-treated EAE | 10 | 361.70 ± 106.990 |  | WT vs Treg-treated EAE | -55.200 | 46.951 | 0.750 |
|  | Treg-treated EAE | 10 | 487.60 ± 115.721 |  | Sham-treated EAE vs Treg-treated EAE | -125.900 | 46.951 | 0.037 |
|  | *SS* | 79654.467 | |  |  |  |  |  |
|  | *df* | 2 | |  |  |  |  |  |
|  | *MS* | 39827.233 | |  |  |  |  |  |
|  | *F* | 3.613 | |  |  |  |  |  |
|  | *P* | 0.041 | |  |  |  |  |  |
| CCN3^+^Olig2^+^ cells/mm^2^ | WT | 10 | 295.50 ± 80.401 |  | WT vs Sham-treated EAE | 17.000 | 38.585 | >0.999 |
|  | Sham-treated EAE | 10 | 278.50± 73.366 |  | WT vs Treg-treated EAE | -68.900 | 38.585 | 0.256 |
|  | Treg-treated EAE | 10 | 364.40 ± 102.397 |  | Sham-treated EAE vs Treg-treated EAE | -85.900 | 38.585 | 0.104 |
|  | *SS* | 41383.400 | |  |  |  |  |  |
|  | *df* | 2 | |  |  |  |  |  |
|  | *MS* | 20691.700 | |  |  |  |  |  |
|  | *F* | 2.780 | |  |  |  |  |  |
|  | *P* | 0.080 | |  |  |  |  |  |
| CCN3^+^Olig2^+^/Olig2^+^ cells (%) | WT | 10 | 21.44 ± 4.940 |  | WT vs Sham-treated EAE | 4.070 | 2.310 | 0.268 |
|  | Sham-treated EAE | 10 | 17.37 ± 4.758 |  | WT vs Treg-treated EAE | -4.282 | 2.310 | 0.224 |
|  | Treg-treated EAE | 10 | 25.72 ± 5.742 |  | Sham-treated EAE vs Treg-treated EAE | -8.352 | 2.10 | 0.004 |
|  | *SS* | 348.842 | |  |  |  |  |  |
|  | *df* | 2 | |  |  |  |  |  |
|  | *MS* | 174.421 | |  |  |  |  |  |
|  | *F* | 6.539 | |  |  |  |  |  |
|  | *P* | 0.005 | |  |  |  |  |  |

**Table S11. Kolmogorov-Smirnov test for normality and homogeneity of variance for mRNA levels**

| Group | | Kolmogorov-Smirnov test | | |  | Levene's test | | | |
| --- | --- | --- | --- | --- | --- | --- | --- | --- | --- |
|  |  | *D* | *df* | *P* |  | *F* | *df1* | *df2* | *P* |
| Ifn-γ | WT | 0.183 | 5 | 0.200 |  | 0.600 | 2 | 12 | 0.564 |
|  | Sham-treated EAE | 0.372 | 5 | 0.023 |  |  |  |  |  |
|  | Treg-treated EAE | 0.183 | 5 | 0.200 |  |  |  |  |  |
| Tnf | WT | 0.213 | 5 | 0.200 |  | 0.411 | 2 | 12 | 0.672 |
|  | Sham-treated EAE | 0.371 | 5 | 0.023 |  |  |  |  |  |
|  | Treg-treated EAE | 0.241 | 5 | 0.200 |  |  |  |  |  |
| Il-6 | WT | 0.260 | 5 | 0.200 |  | 0.293 | 2 | 12 | 0.751 |
|  | Sham-treated EAE | 0.451 | 5 | 0.001 |  |  |  |  |  |
|  | Treg-treated EAE | 0.316 | 5 | 0.115 |  |  |  |  |  |
| Il-10 | WT | 0.178 | 5 | 0.200 |  | 3.185 | 2 | 12 | 0.078 |
|  | Sham-treated EAE | 0.218 | 5 | 0.200 |  |  |  |  |  |
|  | Treg-treated EAE | 0.239 | 5 | 0.200 |  |  |  |  |  |
| Ccn3 | WT | 0.198 | 5 | 0.200 |  | 5.678 | 2 | 12 | 0.018 |
|  | Sham-treated EAE | 0.253 | 5 | 0.200 |  |  |  |  |  |
|  | Treg-treated EAE | 0.252 | 5 | 0.200 |  |  |  |  |  |

**Table S12. One-way ANOVA analysis for Il-10 level**

| Group | Description | |  | Multiple Comparison | | | |
| --- | --- | --- | --- | --- | --- | --- | --- |
|  | *N* | *Mean ± SD* |  | **Groups** | *Mean Range* | *SE* | *P* |
| WT | 5 | 1.01 ± 0.19 |  | WT vs Sham-treated EAE | 0.028 | 0.090 | >0.999 |
| Sham-treated EAE | 5 | 0.99 ± 0.05 |  | WT vs Treg-treated EAE | -0.028 | 0.090 | >0.999 |
| Treg-treated EAE | 5 | 1.04 ± 0.15 |  | Sham-treated EAE vs Treg-treated EAE | -0.056 | 0.090 | >0.999 |
| *SS* | 0.008 | |  |  |  |  |  |
| *df* | 2 | |  |  |  |  |  |
| *MS* | 0.007 | |  |  |  |  |  |
| *F* | 0.191 | |  |  |  |  |  |
| *P* | 0.829 | |  |  |  |  |  |

**Table S13. Nonparametric test-Kruskal-Wallis test has been performed for Ifn-γ, Ccn3, Il-6 and Tnf mRNA levels**

| Gene | Group | Description | | |  | Multiple Comparison | | | |
| --- | --- | --- | --- | --- | --- | --- | --- | --- | --- |
|  |  | *N* | *Median* | *IQR* |  | *Groups* | *H* | *SE* | *P^a^* |
| Ifn-γ | WT | 5 | 1.01 | 0.32 |  | WT vs Sham-treated EAE | -8.500 | 2.286 | 0.008 |
|  | Sham-treated EAE | 5 | 1.98 | 0.75 |  | WT vs Treg-treated EAE | -6.500 | 2.286 | 0.064 |
|  | Treg-treated EAE | 5 | 1.73 | 0.86 |  | Treg-treated EAE vs Sham-treated EAE | 2.000 | 2.286 | >0.999 |
|  | *H* | 9.893 | | |  |  |  |  |  |
|  | *df* | 2 | | |  |  |  |  |  |
|  | *P* | 0.007 | | |  |  |  |  |  |
| Ccn3 | WT | 5 | 1.12 | 1.07 |  | Sham-treated EAE vs WT | 2.200 | 2.828 | >0.999 |
|  | Sham-treated EAE | 5 | 0.85 | 0.44 |  | WT vs Treg-treated EAE | -5.200 | 2.828 | 0.198 |
|  | Treg-treated EAE | 5 | 1.96 | 3.10 |  | Sham-treated EAE vs Treg-treated EAE | -7.400 | 2.828 | 0.027 |
|  | *H* | 7.220 | | |  |  |  |  |  |
|  | *df* | 2 | | |  |  |  |  |  |
|  | *P* | 0.027 | | |  |  |  |  |  |
| Il-6 | WT | 5 | 1.37 | 0.95 |  |  |  |  |  |
|  | Sham-treated EAE | 5 | 0.39 | 0.62 |  |  |  |  |  |
|  | Treg-treated EAE | 5 | 0.84 | 0.49 |  |  |  |  |  |
|  | *H* | 2.540 | | |  |  |  |  |  |
|  | *df* | 2 | | |  |  |  |  |  |
|  | *P* | 0.281 | | |  |  |  |  |  |
| Tnf | WT | 5 | 1.12 | 0.53 |  |  |  |  |  |
|  | Sham-treated EAE | 5 | 1.50 | 1.10 |  |  |  |  |  |
|  | Treg-treated EAE | 5 | 1.44 | 0.65 |  |  |  |  |  |
|  | *H* | 5.154 | | |  |  |  |  |  |
|  | *df* | 2 | | |  |  |  |  |  |
|  | *P* | 0.076 | | |  |  |  |  |  |

a: Multiple comparisons have been adjusted by Bonferroni.

**Figure** **Captions**

**Fig. S1** **Mice developed experimental autoimmune encephalomyelitis (EAE) symptoms on 9 days post immunization.** Changes in (**A**) disease severity scores and (**B**) body weight were observed in the experimental autoimmune encephalomyelitis (EAE) (n = 53) and sham groups (n = 10). All the data are presented as the median ± interquartile range.

**Figures**

**Fig. S1**


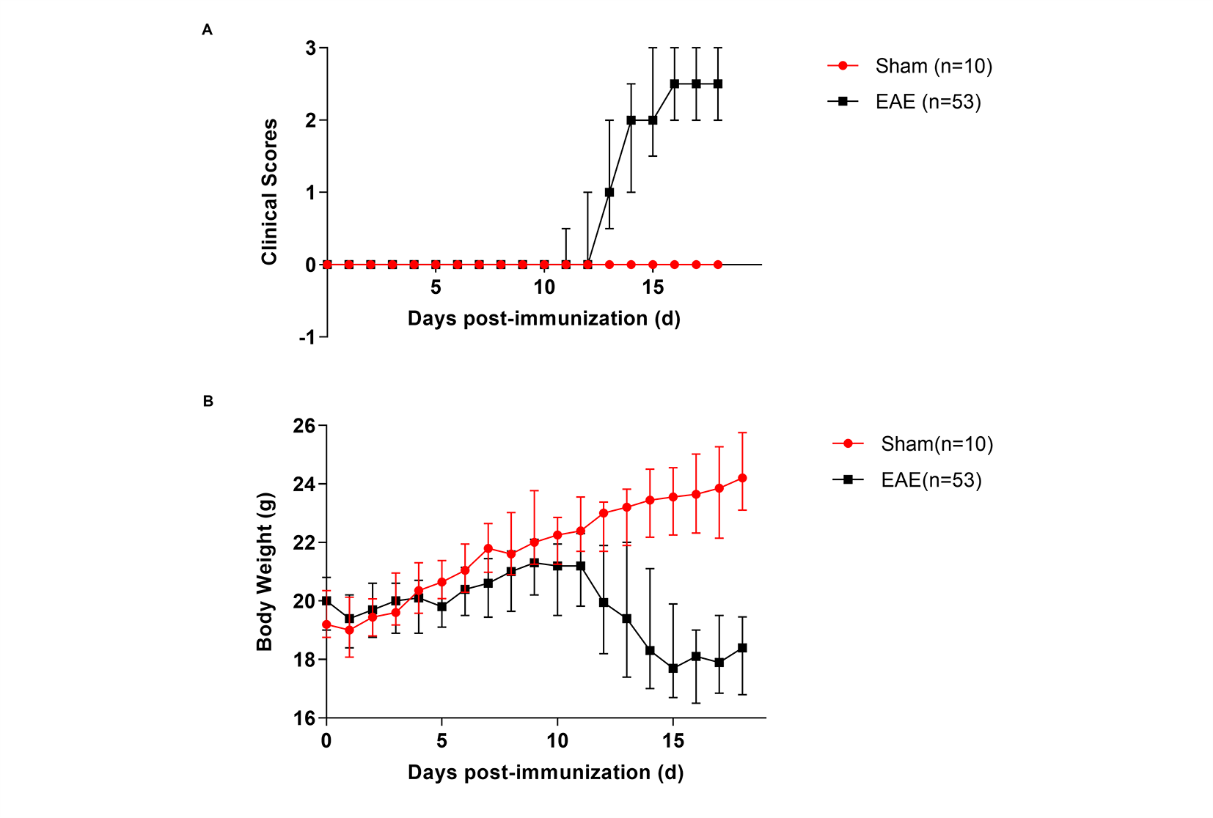

Supplement: Supplementary file 1 — Supplementary Information. [file 41598_2024_56739_MOESM1_ESM.docx]
